# Protein quality control machinery supports primary ciliogenesis by eliminating GDP-bound Rab8-family GTPases

**DOI:** 10.1016/j.isci.2023.106652

**Published:** 2023-04-10

**Authors:** Toshiki Takahashi, Jun Shirai, Miyo Matsuda, Sae Nakanaga, Shin Matsushita, Kei Wakita, Mizuki Hayashishita, Rigel Suzuki, Aya Noguchi, Naoto Yokota, Hiroyuki Kawahara

**Affiliations:** 1 Laboratory of Cell Biology and Biochemistry, Department of Biological Sciences, Tokyo Metropolitan University, Tokyo 192-0397, Japan

**Keywords:** Molecular biology, Cell biology

## Abstract

The small GTPase Rab8 plays a vital role in the vesicular trafficking of cargo proteins from the *trans-*Golgi network to target membranes. Upon reaching its target destination, Rab8 is released from the vesicular membrane into the cytoplasm via guanosine triphosphate (GTP) hydrolysis. The fate of GDP-bound Rab8 released from the destination membranes, however, has not been investigated adequately. In this study, we found that GDP-bound Rab8 subfamily proteins are targeted for immediate degradation, and the pre-emptive quality control machinery is responsible for eliminating these proteins in a nucleotide-specific manner. We provide evidence that components of this quality control machinery have a critical role in vesicular trafficking events, including the formation of primary cilia, a process regulated by the Rab8 subfamily. These results suggest that the protein degradation machinery plays a critical role in the integrity of membrane trafficking by limiting the excessive accumulation of GDP-bound Rab8 subfamily proteins.

## Highlights

• GDP-bound Rab8 subfamily proteins are polyubiquitinated for degradation
• UBQLN4 is required for Rab8a degradation in a nucleotide-specific manner
• RNF126 plays a critical role in the degradation of GDP-bound Rab8a
• The protein degradation machinery is necessary for primary cilium formation

## Graphical abstract

**Figure:**
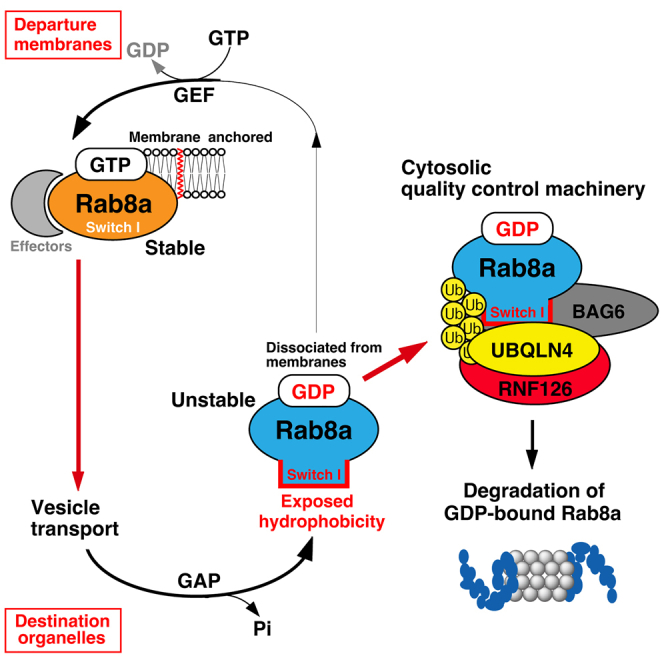

## Introduction

Small monomeric GTPases belonging to the Ras superfamily are molecular switches that cycle between their active guanosine triphosphate (GTP)-bound and inactive GDP-bound states.^1^^,^^2^ In the case of cell-membrane-anchored Ras protein, GDP/GTP exchange is stimulated by guanine nucleotide exchange factor (GEF) to transduce proliferative signals in response to extracellular growth stimuli.^3^^,^^4^^,^^5^ In the absence of growth factors, Ras-bound GTP nucleotide is hydrolyzed by the intrinsic GTPase activity of Ras, resulting in its rapid inactivation.^2^ Thus, the activity of Ras is controlled by its reversible nucleotide exchange cycle on the cell membrane.

In contrast to Ras, the Rab family of small GTPases is responsible for membrane vesicular trafficking between cytoplasmic organelles.^6^^,^^7^^,^^8^^,^^9^^,^^10^^,^^11^ Rab8a, for example, plays an important role in the long-distance vesicular trafficking of cargo proteins from the *trans-*Golgi network to target destinations with specific cytoskeletal-filament interactions.^12^^,^^13^^,^^14^^,^^15^^,^^16^^,^^17^^,^^18^^,^^19^^,^^20^ At the end of its journey, GTP hydrolysis facilitates the dissociation of Rab8a from the vesicular membrane to the cytoplasm. It has long been assumed that GDP-bound Rab8a released from the destination membrane is retroverted through cytoplasmic transport (or simple diffusion) to the departure organelles where it is reactivated by a specific GEF.^21^^,^^22^ The cytoplasmic machinery responsible for transporting GDP-bound Rab8a back to the departure membranes, if it exists, is not adequately understood.

The cytoplasmic accumulation of GDP-bound Rab8a has been reported to have deleterious effects on cell vesicular trafficking due to its dominant-negative and aggregation-prone nature.^17^^,^^23^^,^^24^^,^^25^^,^^26^^,^^27^^,^^28^^,^^29^^,^^30^ Indeed, the forced expression of GDP-bound Rab8a inhibits several Rab8a-mediated processes, such as primary ciliogenesis,^17^^,^^31^^,^^32^ insulin-stimulated GLUT4 translocation,^30^^,^^33^ the transformation of macropinosomes into tubules,^17^ and the distribution of RABIN8, a GEF for Rab8a.^34^ These observations suggest that the amount of GDP-bound Rab8a must be maintained at a minimum level. Therefore, it is necessary to understand the fate of GDP-bound Rab8a after its cytoplasmic retrieval. However, the mechanism responsible for maintaining minimum amounts of inactive Rab8a remains to be elucidated.

In a previous study, we proposed that cytoplasmic Rab8a in its GDP-bound form is recognized by BAG6 and targeted for degradation.^35^ In contrast, the vast majority of active GTP-bound Rab8a is resistant to degradation and does not have detectable affinity for BAG6. It is not known how BAG6 selectively determines the stability of GDP-bound Rab8a. BAG6 is a multifunctional protein localized in the cytoplasm and nucleus, and one of its cytoplasmic functions is pre-emptive protein quality control.^36^^,^^37^^,^^38^^,^^39^^,^^40^^,^^41^^,^^42^ This quality control machinery is essential for degrading mislocalized transmembrane domain proteins that fail to be incorporated into the target membrane and thus expose their hydrophobic stretches (such as the transmembrane domain) in the cytoplasm.^36^^,^^38^ Indeed, BAG6 possesses a preference for such exposed hydrophobicity.^37^^,^^38^^,^^43^^,^^44^ The pre-emptive quality control machinery is composed of not only BAG6 but also the RING finger-type ubiquitin ligase RNF126,^45^^,^^46^ and ubiquitin receptor UBQLN4.^47^^,^^48^ Therefore, it is necessary to investigate whether these components of the pre-emptive quality control system are essential for membrane trafficking events by controlling Rab8 subfamily proteins, such as Rab8a and Rab10, in a GDP-bound form-specific manner.

In this study, we focused on this issue by investigating the possible interplay between Rab8 subfamily proteins and pre-emptive quality control components. We found that UBQLN4 predominantly recognizes the GDP-bound forms of Rab8a and Rab10, which are destined for immediate degradation, while their stable GTP-bound counterparts are scarcely recognized. Furthermore, major components of the pre-emptive protein quality control machinery, including RNF126 and BAG6, are all associated with Rab8 subfamily proteins in a nucleotide-dependent manner. UBQLN4 and RNF126 are both necessary for the instability of GDP-bound Rab8a, since their depletion results in the stabilization of GDP-bound species. We also found that the degradation of GDP-bound Rab10 is inhibited by the depletion of BAG6 or RNF126. Furthermore, we demonstrated that deficiencies in the pre-emptive quality control machinery induce defects in primary ciliogenesis, the vesicular trafficking of which is regulated in a redundant manner by Rab8 and Rab10.^49^ Collectively, these results suggest that the ubiquitination machinery responsible for pre-emptive protein quality control prevents the excessive accumulation of GDP-bound Rab8 subfamily proteins, which must be maintained at a minimum level to ensure proper membrane vesicle sorting in the cytoplasm.

## Results

### GDP-bound Rab8 subfamily proteins are unstable in HeLa cells

**Figure 1:**
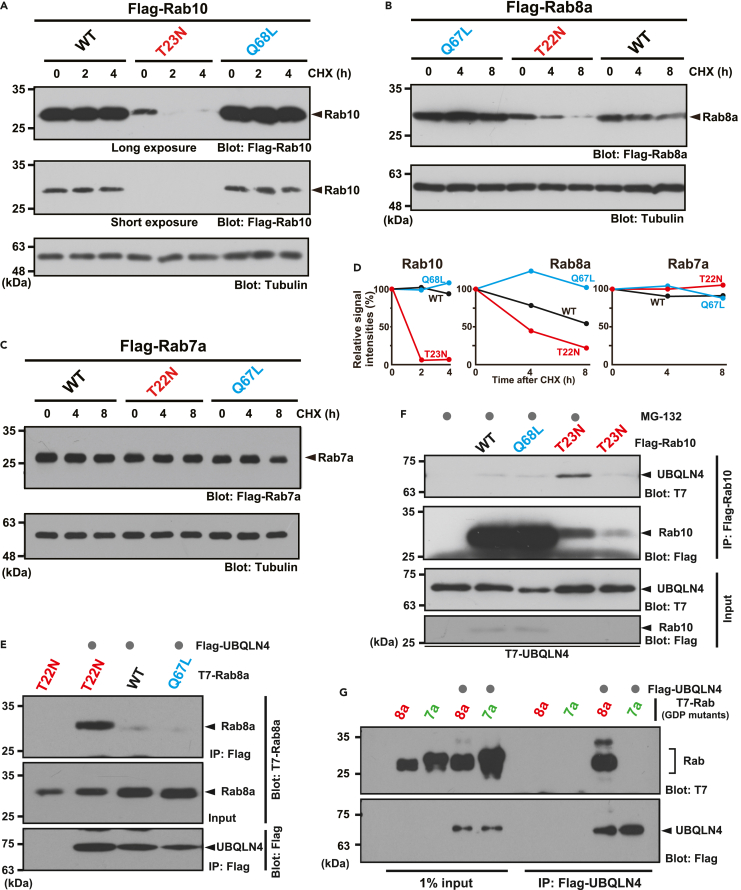
UBQLN4 preferentially interacts with unstable species of Rab family of proteins (A–C) The Rab10-T23N and Rab8a-T22N mutants are inactive forms that dominantly bind to GDP, whereas the Rab10-Q68L and Rab8a-Q67L mutants lack GTPase activity and are constitutively active as the GTP-bound forms. Cycloheximide (CHX) chase experiments show that GDP-bound Rab10-T23N (A) and Rab8a-T22N (B) are short-lived in HeLa cells, while their GTP-bound active mutant and wild-type (WT) proteins are stable (A, B). In contrast, Rab7a is stable even in its GDP-bound T22N mutant form (C). Tubulin was used as a loading control. Note that GDP-bound Rab10-T23N is difficult to express at a level equivalent to that of stable WT Rab10 due to its extreme instability. (D) Graphs indicate the quantified signal intensities of Rab10 (A), Rab8a (B), and Rab7a (C) blots relative to loading controls at the indicated time points after CHX addition. Signals at time zero were defined as 100%. (E and F) Co-immunoprecipitation analysis shows that UBQLN4 efficiently co-precipitates with Rab8a-T22N, but scarcely with Rab8a-Q67L (E). The case was similar for the GDP-locked Rab10-T23N and nucleotide hydrolysis-deficient Rab10-Q68L mutants (F). MG-132 (10 μM) was added to the cell cultures 4 h before harvesting the cells. (G) UBQLN4 co-precipitates with Rab8a-T22N but not Rab7a-T22N. T7-tagged Rab8a and Rab7a in their GDP-bound forms were co-expressed with Flag-UBQLN4 in HeLa cells. The cells were treated with 10 μM MG-132 for 4 h. Flag-precipitates were blotted with anti-T7 and anti-Flag antibodies, respectively.

Rab10, a paralog of Rab8a, was previously shown to bind to BAG6,^35^ and functions redundantly with Rab8 in primary ciliogenesis.^49^ Cycloheximide (CHX) chase experiments suggested that wild-type Rab10 and Rab8a were stable proteins (Figures 1A, 1B, and 1D). However, we found that the GDP-bound Rab10-T23N mutant has a very short half-life in HeLa cells (Figures 1A and 1D), similar to the GDP-bound Rab8a-T22N mutant (Figures 1B and 1D). In contrast, constitutively active GTP-bound Rab10-Q68L and Rab8a-Q67L mutant proteins, which have compromised GTPase activity, were highly stable (Figures 1A, 1B and 1D). These observations suggest that the stability of the Rab8 subfamily of small GTPases is controlled in a nucleotide-specific manner.

In contrast to Rab8 subfamily proteins, Rab7a was identified as a small GTPase with a non-detectable affinity for BAG6.^35^ We found that Rab7a was highly stable in HeLa cells, even in its GDP-bound T22N mutant form (Figures 1C and 1D). These results suggest that the instability of GDP-bound Rab proteins is subfamily specific and apparently correlates with their affinity for BAG6.

### UBQLN4 is required for Rab8a degradation in a nucleotide-specific manner

Because UBQLN4 is a critical ubiquitin receptor for pre-emptive quality control,^47^^,^^48^ we examined whether UBQLN4 has an affinity for Rab8 subfamily proteins. We found that GDP-bound Rab8a-T22N co-immunoprecipitated with UBQLN4 in HeLa cell extracts (Figure 1E). In contrast, constitutively active Rab8a-Q67L did not co-precipitate with UBQLN4 under identical conditions (Figure 1E). Similarly, GDP-bound Rab10-T23N co-immunoprecipitated with UBQLN4, whereas constitutively active Rab10-Q68L did not (Figure 1F), even though Rab10-Q68L accumulates in much larger amounts than Rab10-T23N due to their difference in stability. Notably, the highly stable Rab7a protein showed little affinity for UBQLN4 (Figure 1G), even in its GDP-bound form. These observations suggest a clear preference of UBQLN4 for unstable Rab proteins, and their interactions with UBQLN4 are nucleotide-specific.

**Figure 2:**
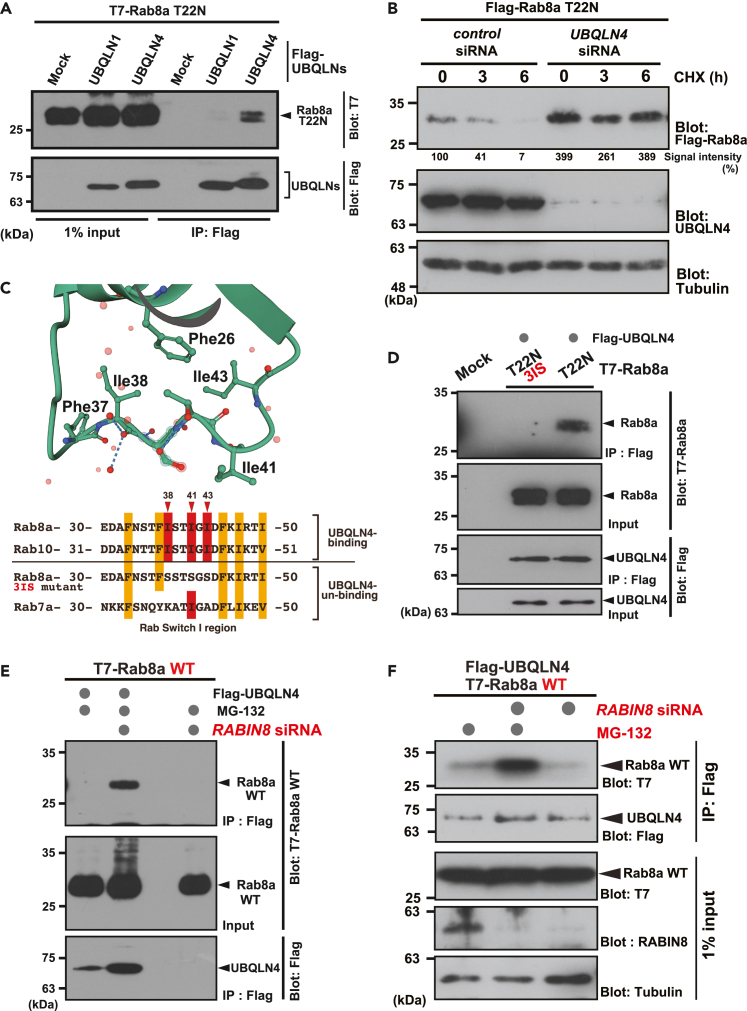
UBQLN4 is essential for the degradation of Rab8a in a nucleotide-specific manner (A) UBQLN4 dominantly co-precipitates with Rab8a-T22N over UBQLN1. Flag-tagged UBQLN1 or UBQLN4 was precipitated with T7-tagged Rab8a-T22N in HeLa cell extracts and probed with the indicated antibodies. All cells were treated with 10 μM MG-132 for 4 h before harvesting. See also Figure S1. (B) *UBQLN4* knockdown blocked the degradation of GDP-bound Rab8a. The quantified signal intensities of Rab8a-T22N protein relative to loading controls at the indicated time points were noted at the bottom of the blot panel. The signal at time zero in control siRNA-treated cells was defined as 100%. (C and D) Hydrophobic residues in the Switch I region of Rab8a are critical for UBQLN4 recognition. Conformations of the Rab8a Switch I loop in its GDP-bound form (C, upper panel, 4LHV). The hydrophobic residues of the Rab8a Switch I region are exposed in its GDP-bound form. Amino acid sequence alignments of the Switch I region in Rab8a and Rab7a (C, lower panel). Three conserved hydrophobic residues and the other hydrophobic residues in the Rab8 subfamily are indicated in red and orange, respectively. The three isoleucine residues in Rab8a Switch I were substituted with serine (T22N-3IS mutant). The hydrophobic residues Ile38 and Ile43 of Rab8a are not conserved in Rab7a. The Rab8a-T22N-3IS mutant lost its affinity for the UBQLN4 protein (D). Mock indicates cells with empty vector transfection as a negative control. (E and F) Depletion of the Rab8a GEF, RABIN8, enhanced the physical interaction between wild-type (WT) Rab8a and UBQLN4 (E). T7-tagged WT Rab8a and Flag-tagged UBQLN4 were co-expressed in *RABIN8* siRNA-treated cells and the Flag immunoprecipitates were probed with an anti-T7 antibody. Cells were treated with or without 10 μM MG-132 for 4 h before harvesting as indicated (F).

A previous study suggested that defective model transmembrane protein substrates for pre-emptive quality control are predominantly recognized by UBQLN4 over UBQLN1, which is a UBQLN4-related ubiquitin-like (UBL) and ubiquitin-associated (UBA) domain protein.^47^^,^^50^ To determine the specificity of UBQLN family proteins for GDP-bound Rab8a, we compared the binding of UBQLN4 and UBQLN1 to Rab8a-T22N. As shown in Figure 2A, UBQLN4 co-immunoprecipitated with Rab8a-T22N more effectively than UBQLN1, suggesting that UBQLN4 possesses a higher affinity for inactive species of Rab8a. In accordance with this observation, the STI-II region, which determines the substrate specificity of UBQLN4,^47^ is necessary for the recognition of Rab8a-T22N (Figures S1A and S1B).

Because UBQLN4 was found to be associated with Rab8a-T22N, we examined the possible role of UBQLN4 in the instability of GDP-bound Rab8a. We found that *UBQLN4* knockdown effectively blocked the degradation of Rab8a-T22N (Figure 2B), suggesting that endogenous UBQLN4 is essential for the degradation of GDP-bound Rab8a.

UBQLN4 recognizes the exposed hydrophobic residues of client proteins and targets them for degradation.^47^ Therefore, we suspected that UBQLN4 could recognize the exposed hydrophobic residues of GDP-bound Rab8a upon its release from membranes. Previous studies suggested that hydrophobic residues in the Switch I loop are exposed to the cytoplasmic surface when Rab8a is bound to guanosine diphosphate (GDP) (Figure 2C, upper panel).^51^^,^^52^ To examine whether this exposed hydrophobicity in the GTPase domain is important for UBQLN4 recognition, we used the 3IS mutant, in which three hydrophobic residues in the Switch I region were substituted with hydrophilic serine residues (Figure 2C, lower panel). We found that this highly stable Rab8a-T22N-3IS mutant^35^ failed to co-immunoprecipitate with UBQLN4 (Figure 2D). The interaction between UBQLN4 and Rab8a also depends on ubiquitination events, given that treatment with TAK-243 (MLN7243), a small molecule inhibitor of the ubiquitin-activating enzyme E1, weakened the co-precipitation of Rab8a-T22N with UBQLN4 (Figure S1C). Collectively, these findings indicate that UBQLN4 targets Rab8a for degradation in a nucleotide- and ubiquitination-dependent manner by recognizing exposed hydrophobic stretches in the GTPase domain.

### The GEF substrate portion of wild-type Rab8a is unstable for proteasomal degradation

To examine whether the nucleotide exchange of wild-type Rab8a is indeed critical for UBQLN4 recognition, we depleted the endogenous GEF for Rab8a, RABIN8/RAB3IP.^52^^,^^53^ This depletion should theoretically lock the GDP/GTP exchange cycle of wild-type Rab8a in the GDP-bound state, thereby causing the cytoplasmic accumulation of GDP-bound species. In accordance with this idea, the co-precipitation of UBQLN4 with wild-type Rab8a was stimulated by *RABIN8* knockdown (Figure 2E). Importantly, the amount of wild-type Rab8a that co-precipitated with UBQLN4 under GEF suppression was greatly augmented after treatment with MG-132, a proteasome inhibitor (Figure 2F), even though the total amount of wild-type Rab8a was insensitive to MG-132. These observations suggest that a cryptic portion of the GEF substrate (a GDP-bound species of wild-type Rab8a) is an unstable protein targeted by UBQLN4 for immediate degradation.

### GDP-bound cryptic species of Rab8a is polyubiquitinated

**Figure 3:**
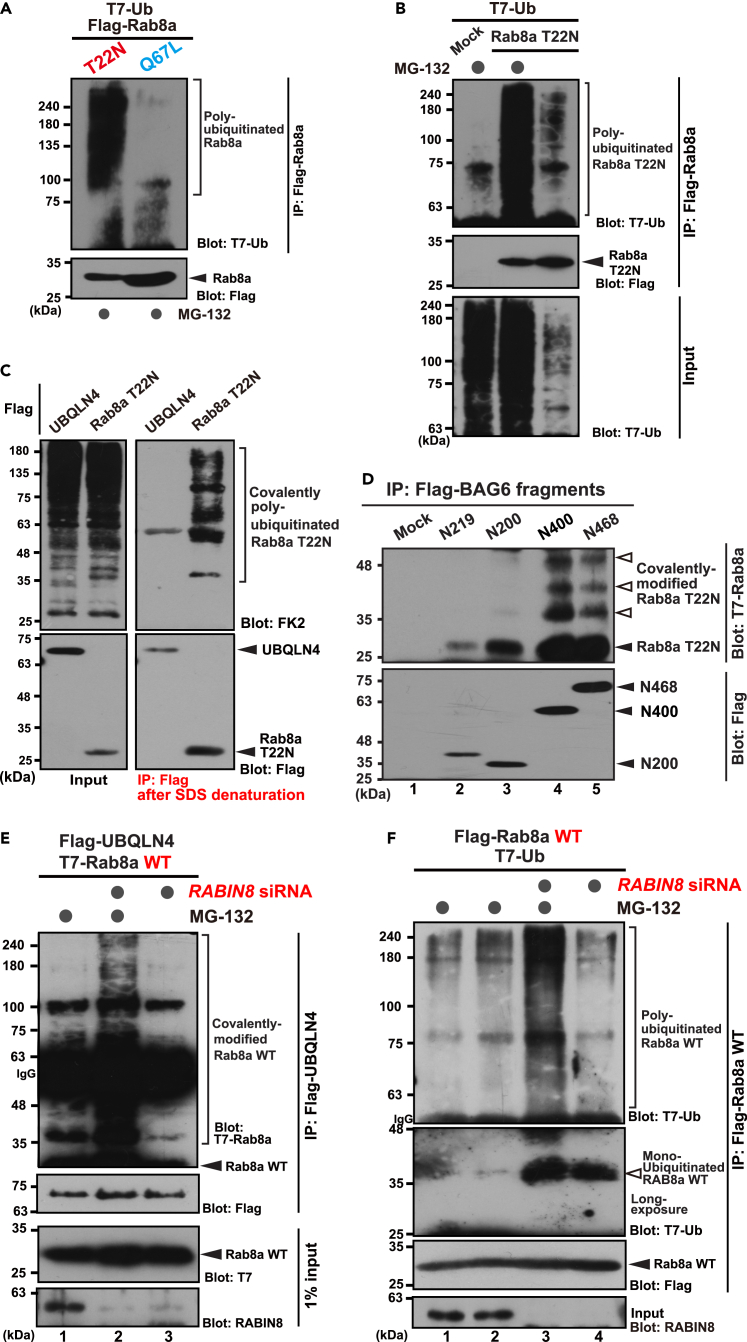
GDP-bound Rab8a is polyubiquitinated (A) Rab8a-T22N is polyubiquitinated. Flag-tagged T22N and Q67L Rab8a mutants were co-expressed with T7-tagged ubiquitin in HeLa cells treated with 10 μM MG-132 for 4 h. Flag-precipitates from the cell lysates were blotted with an anti-T7 antibody to detect the polyubiquitin co-precipitation of Rab8a proteins. (B) Polyubiquitin modification of Rab8a-T22N is MG-132-dependent. (C) Endogenous ubiquitin covalently modifies GDP-bound Rab8a. Before immunoprecipitations, cell extracts were boiled with 1% SDS to denature Rab8a-T22N, and then diluted. Flag-tagged Rab8a-T22N was immunoprecipitated and probed with an anti-polyubiquitin FK2 antibody to detect covalent ubiquitin conjugation on Rab8a-T22N. UBQLN4 was used as a negative control for direct ubiquitination. (D) Flag-tagged BAG6 fragments and T7-tagged Rab8a-T22N were co-immunoprecipitated by anti-Flag antibody and probed with an anti-T7 antibody to detect co-precipitated Rab8a-T22N. Prolonged exposure of T7 blots with N400 or N468 precipitates shows multiple ladder-like signals at approximately 8 kDa intervals (indicated by white arrowheads). N219, N200, and N468 stand for N-terminal 219, 200, and 468 residues fragments of BAG6, respectively, and N400 stands for a tandemly fused fragment of N200. “Mock” indicates empty vector transfection as a negative control. (E) Covalent modifications of wild-type (WT) Rab8a are enhanced following depletion of *RABIN8*, a GEF for Rab8a. T7-tagged WT Rab8a was co-expressed with Flag-UBQLN4 in *RABIN8* siRNA-treated cells, and Flag-immunoprecipitates were probed with an anti-T7 antibody. High molecular weight covalent modifications of wild-type (WT) Rab8a co-precipitated with UBQLN4 are evident in *RABIN8*-depleted cells (upper panel, compare lanes 1 and 2). The modifications are MG-132-dependent (compare lanes 2 and 3). IgG indicates immunoglobulin signal. (F) Polyubiquitin modifications of WT Rab8a are enhanced following depletion of *RABIN8*. Flag-tagged WT Rab8a was co-expressed with T7-tagged ubiquitin with or without *RABIN8* depletion in HeLa cells, and Flag-immunoprecipitates were probed with anti-T7 antibody. Enhanced polyubiquitin modifications of WT Rab8a are evident in *RABIN8*-depleted cells (upper panel, compare lanes 2 and 3). Also note that MG-132 treatment enhanced polyubiquitination of Rab8a (upper panel, compare lanes 3 and 4). Prolonged exposure of the blot shows prominent mono-ubiquitinated signals of wild-type (WT) Rab8a, only under the condition of *RABIN8* depletion (indicated by a white arrowhead, lanes 3 and 4 in the second panel). Cells were treated with 10 μM MG-132 for 4 h before harvesting as indicated. IgG indicates immunoglobulin signal.

Given that UBQLN4 is known to function as a ubiquitin receptor for proteasome-dependent protein degradation,^47^^,^^48^ we speculated that GDP-bound Rab8a, a UBQLN4 client, might be polyubiquitinated. We co-expressed T7-tagged ubiquitin with Rab8a-T22N mutant protein in HeLa cells to examine this possibility. Strikingly, Rab8a-T22N massively co-immunoprecipitated with T7-tagged polyubiquitin, while highly stable GTP-bound Rab8a-Q67L did not (Figure 3A). Polyubiquitination of Rab8a-T22N was enhanced following MG-132 treatment (Figure 3B), suggesting that polyubiquitinated Rab8a-T22N is a proteasomal substrate for degradation. In accordance with this idea, SDS-mediated denaturation before immunoprecipitation did not abolish the co-precipitation of endogenous polyubiquitin chains with Rab8a-T22N (Figure 3C). Furthermore, co-precipitation of Rab8a-T22N with N-terminal fragments of BAG6 showed multiple ladder-like signals of Rab8a at approximately 8-kDa intervals (Figure 3D, lanes 4 and 5, indicated by white arrowheads). These observations suggest the presence of covalent modifications of Rab8a-T22N with polyubiquitin on this chaperone-like protein.

Rab8a polyubiquitination was observed not only in the T22N GDP-bound mutant but also in its wild-type form following Rab8a-specific GEF knockdown. Although only a trace amount of polyubiquitin signals co-precipitated with wild-type Rab8a (Figure 3E, lane 1; Figure 3F, lane 1), Rab8a-GEF depletion augmented the signals of covalent modifications of wild-type Rab8a that co-precipitated with UBQLN4 (Figure 3E, smear signals in lane 2). In accordance with this observation, co-precipitation of T7-tagged ubiquitin with wild-type Rab8a was stimulated with *RABIN8* knockdown (Figure 3F, upper panel; compare lanes 2 and 3). These signals of wild-type Rab8a were not detected in the absence of MG-132 (Figure 3F; compare lanes 3 and 4), suggesting that the polyubiquitinated species induced by RABIN8 depletion are proteasomal substrates. Intriguingly, RABIN8 depletion also stimulates mono-ubiquitination of Rab8a WT at 35-kDa irrespective of MG-132 addition (Figure 3F, second panel, indicated by a white arrowhead). These observations collectively support the notion that native Rab8a can be ubiquitinated in a GDP-bound species-specific manner.

### RNF126 ubiquitin ligase is essential for the degradation of GDP-bound Rab8a

The identification of the ubiquitination machinery responsible for the targeted degradation of Rab8a proteins in a nucleotide-dependent manner is crucial for understanding the novel regulatory mechanism of this small GTPase protein. RNF126 was identified as a BAG6-associated RING finger E3 ligase that is critical for the pre-emptive quality control of mislocalized prion proteins.^45^ Therefore, we investigated whether RNF126 has a physical affinity for GDP-bound Rab8a targeted for degradation.

**Figure 4:**
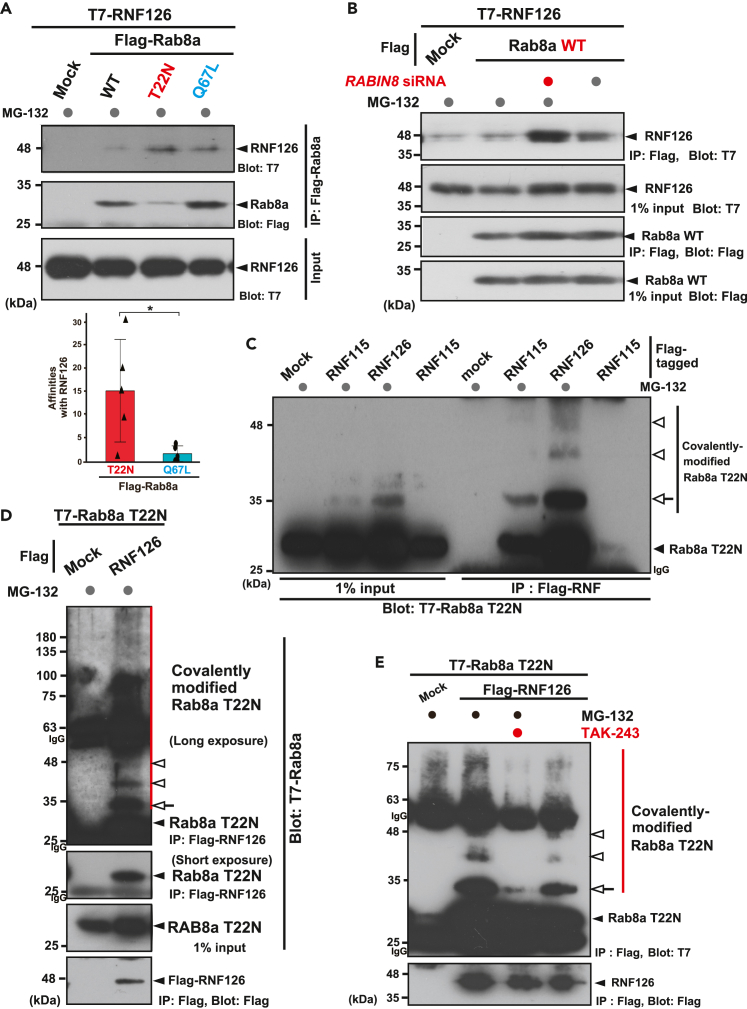
Rab8a co-precipitates with RNF126 in a nucleotide-specific manner (A) T7-tagged RNF126 was preferentially co-precipitated with Flag-tagged Rab8a-T22N, and less efficiently co-precipitated with Rab8a-Q67L, a constitutively active mutant. MG-132 (10 μM) was added to the cell culture 4 h before harvesting. Note that the T22N mutant Rab8a was expressed at lower levels than either WT or Q67L (see Flag-blot panel). Graph shows the relative signal intensities of co-precipitated T7-RNF126 with Flag-Rab8a (signal intensities of T7 blots were divided by that of Flag blots). The signal of WT was defined as 1.0. Data are presented as means ± standard deviation (SD) from five independent experiments. ∗p < 0.05 (Student’s *t* test). See also Figure S2A. (B) Enhanced physical interaction was observed between wild-type (WT) Rab8a and RNF126 following RABIN8 depletion (top panel), even though the total amount of WT Rab8a did not change (second panel). MG-132 treatment (10 μM) enhanced WT Rab8a binding with RNF126 under *RABIN8* siRNA condition. (C and D) Multiple ladder-like signals of Rab8a-T22N were co-precipitated with RNF126. T7-tagged Rab8a-T22N was co-precipitated with Flag-tagged RNF126 and its paralog RNF115. The T7-blot in Flag-precipitates showed an additional band of co-precipitated Rab8a-T22N at approximately 35-kDa (indicated as a white arrow). Prolonged exposure of T7 blots showed multiple ladder-like signals at approximately 8-kDa intervals (indicated by white arrowheads) and high molecular weight smear signals (indicated as red vertical bars), suggesting that covalent modifications of Rab8a-T22N occurred in Flag-RNF126 precipitates. Cells were treated with 10 μM MG-132 for 4 h before harvesting as indicated. IgG indicates immunoglobulin signals. See also Figure S2B. (E) Covalent modifications of Rab8a-T22N (indicated by white arrowheads and an arrow) co-precipitated with RNF126 were abolished by 4 h treatment with 10 μM TAK-243 (MLN7243), an inhibitor of the ubiquitin-activating E1 enzyme. TAK-243 (MLN7243) was added to the cell cultures at 10 μM for 4 h before cell harvest.^54^ IgG indicates immunoglobulin signals. See also Figure S2C.

We found that RNF126 co-precipitated with GDP-bound Rab8a-T22N more efficiently than its GTP-bound Q67L counterpart (Figure 4A). This was also observed for RNF115, an RNF126-related RING finger family protein (Figure S2A) that is also a BAG6-associated E3 ubiquitin ligase.^45^ Collectively, these results show that the major components of the pre-emptive quality control machinery, including RNF126 family E3 ligases, UBQLN4, and BAG6, are all associated with GDP-bound inactive Rab8a in the cytoplasm.

Similar to UBQLN4, the co-immunoprecipitation of RNF126 with wild-type Rab8a was enhanced following the depletion of RABIN8, an endogenous GEF (Figure 4B). Furthermore, the amount of wild-type Rab8a that co-precipitated with RNF126 was augmented by MG-132 treatment with GEF suppression (Figure 4B). These results correspond to the observation that RABIN8 depletion stimulated the polyubiquitination of wild-type Rab8a (Figures 3E and 3F), suggesting that proteasomes degrade a cryptic (GDP-bound) portion of wild-type Rab8a in a nucleotide-dependent manner.

When flag-tagged RNF126 was immunoprecipitated, we noticed that co-precipitated Rab8a-T22N showed multiple ladder-like signals at approximately 8-kDa intervals (Figures 4C and 4D, indicated by white arrowheads and an arrow, see also S2B). Prolonged exposure of the Rab8a blot revealed high-molecular-weight smear signals of Rab8a (Figure 4D, a right lane, indicated by a red line), which were not visible in the control precipitates (Figure 4D, a left lane). These multiple ladder-like signals were abolished by treatment with TAK-243 (Figures 4E and S2C, indicated by white arrowheads and an arrow). These observations suggest that RNF126 is associated with covalently ubiquitinated Rab8a species.

**Figure 5:**
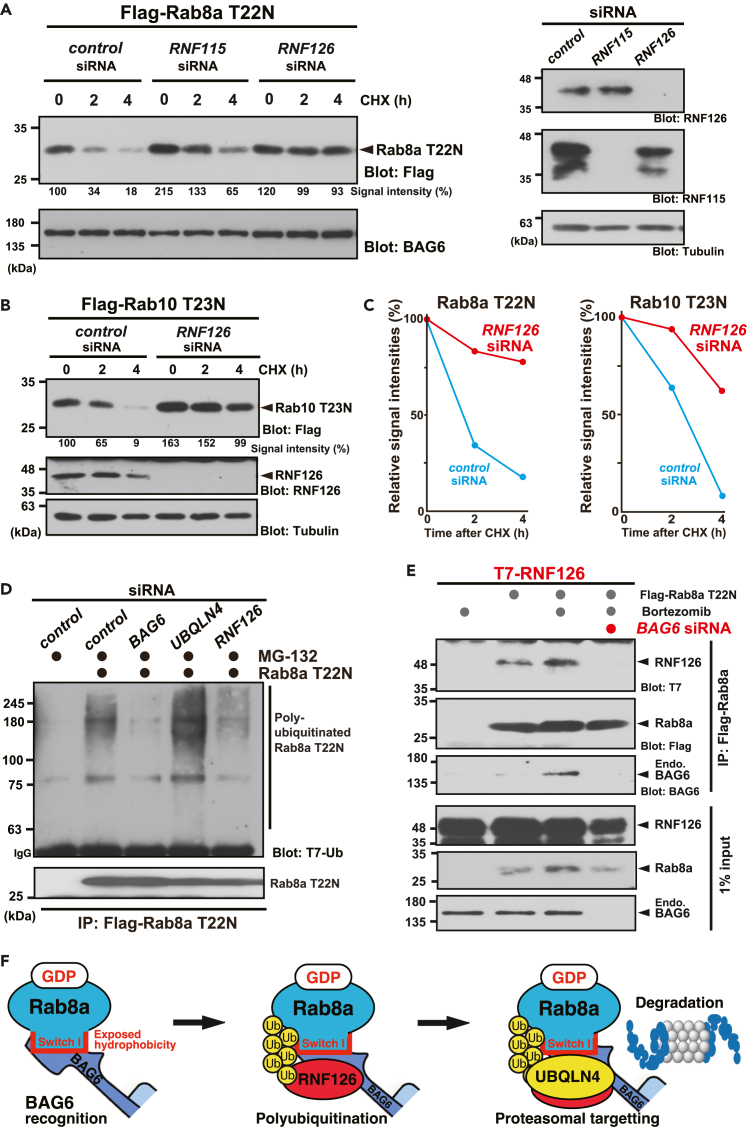
Endogenous BAG6-associated E3 ligases are required for the degradation of GDP-bound Rab8 subfamily proteins (A and B) HeLa cells were transfected with 5 nM siRNA duplexes for *RNF126*, *RNF115,* or *control* siRNA. At 24 h after siRNA transfection, Flag-tagged Rab8a-T22N (A) or Flag-tagged-Rab10-T23N (B) were expressed in the cells. At 24 h after transfection with Rab-expression vectors, the cells were chased with 20 μg/mL cycloheximide (CHX) and harvested at the indicated time after CHX addition. The depletion efficacy and specificity of double-stranded RNAs (*RNF126* and *RNF115* siRNAs) in HeLa cells were verified by western blots with respective antibodies (A, right panel). Endogenous BAG6 or tubulin was used as loading controls. Quantified signal intensities of Rab8a (A) and Rab10 (B) protein blots at the indicated time points relative to loading controls were noted at the bottom of the blot panels. The signal at time zero in control siRNA-treated cells was defined as 100%. (C) The graphs indicate the quantified signal intensities of the Rab8a-T22N (A) and Rab10-T23N (B) protein blots relative to loading controls in RNF126-depleted and control-depleted cells at the indicated time points after CHX addition. Signals at time zero were defined as 100%. See also Figure S3D. (D) Polyubiquitin modifications of immunoprecipitated Rab8a-T22N were suppressed by *BAG6* knockdown, but were enhanced by *UBQLN4* knockdown. All cells were treated with 10 μM MG-132 before harvesting. Note that RNF126 depletion did not completely suppress polyubiquitination of Rab8a-T22N, suggesting that one or more other E3 ligases may collaboratively participate in this process. IgG indicates an immunoglobulin signal. (E) Co-precipitation of T7-RNF126 with Flag-Rab8a-T22N was abolished by BAG6 siRNA. Note that the interaction of RNF126 with Rab8a-T22N was strengthened by bortezomib. (F) Schematic images of recognition, polyubiquitination, and subsequent proteasomal targeting of GDP-bound Rab8a-mediated by BAG6, RNF126, and UBQLN4, respectively.

To investigate the impact of RNF126 ubiquitin ligase on the instability of GDP-bound Rab8a, we performed depletion experiments with a series of specific siRNAs. Cycloheximide-chase experiments showed that knockdown of *RNF126* stabilized Rab8a-T22N (Figures 5A, 5C and S3D), similar to the case in *BAG6* knockdown (Figures S3A and S3C). GDP-bound Rab10 was also stabilized by knockdown of *RNF126* (Figures 5B and 5C) and *BAG6* (Figures S3B and S3C), even though GDP-bound Rab10-T23N is highly unstable (Figures 5B and 5C, *control* siRNA signals). These results suggest that RNF126 is necessary for the elimination of GDP-bound Rab8-family proteins.

Although depletions of BAG6, UBQLN4, and RNF126 all resulted in stabilization of GDP-bound Rab8-family proteins, the polyubiquitination state for Rab8a-T22N seems to be different. As shown in Figure 5D, polyubiquitin modifications of Rab8a-T22N were suppressed by depletion of endogenous BAG6, whereas UBQLN4 depletion enhanced the accumulation of polyubiquitinated Rab8a-T22N. These observations suggest that BAG6 is required for the recognition of Rab8a-T22N before its polyubiquitination (Figure 5F), and that UBQLN4 is critical for proteasomal targeting of polyubiquitinated Rab8a-T22N (Figure 5F). The interaction between RNF126 and GDP-bound Rab8a is dependent on BAG6 (Figure 5E), which might explain why BAG6 depletion blocked the polyubiquitination of Rab8a-T22N. We observed that RNF126 depletion did not completely suppress polyubiquitination of Rab8a-T22N, suggesting that one or more other E3 ligases may collaboratively participate in this process.

### Accumulation of GDP-bound species of wild-type Rab8a in RNF126-depleted cells

**Figure 6:**
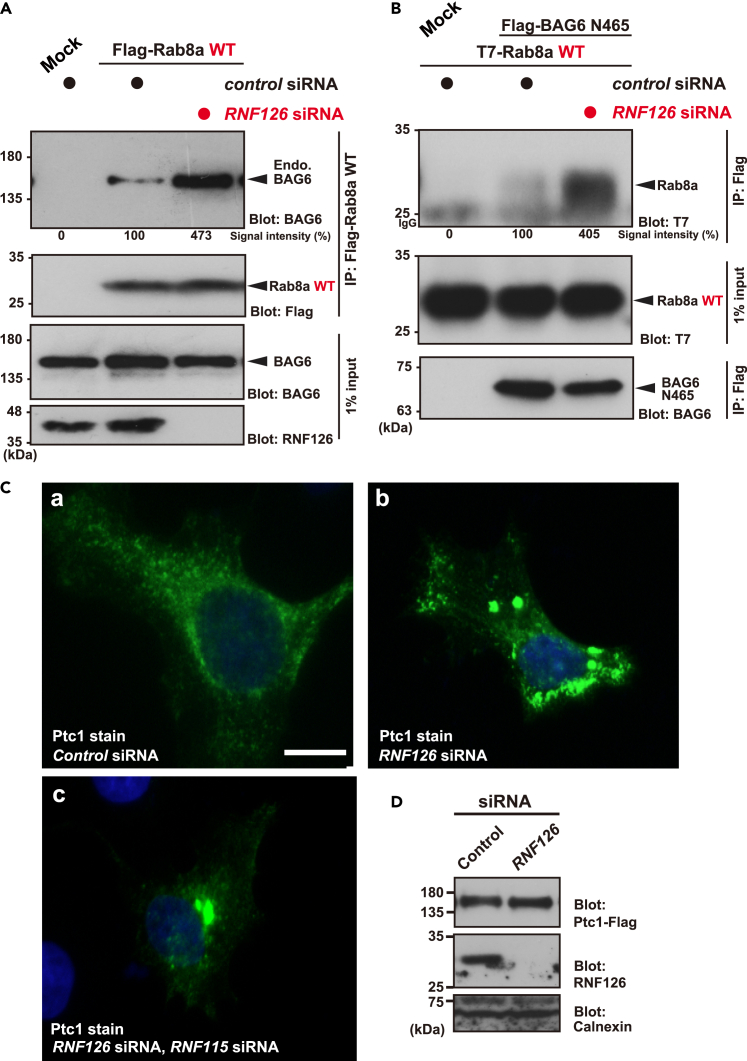
Defective distribution of the endosomal protein Ptc1 in *RNF126*-depleted HeLa cells (A and B) Increased accumulation of wild-type (WT) Rab8a cryptic species, which can be trapped by BAG6 fragments (a probe for GDP-bound Rab8a), was observed in *RNF126* siRNA-treated HeLa cells. Flag-tagged WT Rab8a was immunoprecipitated and probed with BAG6 antibody (A). Similarly, BAG6 N465 fragment was immunoprecipitated and probed with an antibody that recognizes WT Rab8a (B). In both cases, Rab8a-BAG6 co-precipitations were strengthened by RNF126 depletion. The middle panel shows that the total amount of WT Rab8a was unchanged in RNF126-depleted cells. Quantified signal intensities of BAG6 (A) and Rab8a (B) blots were noted at the bottom of the top blot panels. The signals of control siRNA-treated cells were defined as 100%, while those of mock expressions were defined as 0%. (C) Defective distribution of the Ptc1 protein in RNF126/RNF115-depleted cells. At 72 h after transfection with siRNA duplexes (5 nM each), the intracellular distribution of Patched1 (Ptc1)-Flag was examined (shown in green). Nuclei were stained with Hoechst 33258 (shown in blue). Ptc1 distributions in *control* siRNA (a), *RNF126* siRNA (b), *RNF126*/*RNF115* double depletion (c). Note that we used a pCI-neo-based modified expression plasmid with largely compromised promoter activity (by partially deleting the promoter region) to keep the expression of Flag-tagged Ptc1 protein (Ptc1-Flag) at nearly physiological levels. Scale bar: 10 μm. Note that all images presented in this figure were acquired in an identical set of experiments, and the exposure times of all photographs in this figure were the same. See also Figure S4. (D) The expression level of Ptc1-Flag protein was not severely affected by *RNF126* siRNA. Calnexin was used as a loading control.

To examine whether cryptic GDP-bound species of wild-type Rab8a truly accumulate in RNF126-depleted cells, we need a specific probe for GDP-bound Rab8a. To solve this issue, we took advantage of an exogenously expressed BAG6 fragment as a GDP-form-specific probe, given that BAG6 possesses superior binding affinity to GDP-bound Rab8a.^35^ This GDP-form-specific probe enabled us to detect the enhanced accumulation of inactive species of wild-type Rab8a in the cytoplasm of RNF126-depleted cells (Figures 6A and 6B, upper panels). We confirmed that the total amount of wild-type Rab8a was unchanged with RNF126 depletion (Figures 6A and 6B, second panels). Collectively, the endogenous ubiquitin ligase of the pre-emptive quality control pathway is necessary to selectively limit the excessive accumulation of Rab8 subfamily proteins in their cryptic GDP-bound form.

### Deficiency in RNF126 induces the defective distribution of Patched 1

Because the RNF126 was suggested to play a role in the instability of GDP-bound Rab8a (Figures 5A and 5C), and RNF126 deficiency was found to induce mis-accumulation of GDP-bound Rab8a (Figures 6A and 6B), we were interested in examining whether this ubiquitin ligase is linked with the vesicular trafficking of Rab8 client proteins. Patched 1 (Ptc1) is a sonic hedgehog receptor that predominantly localizes in cytoplasmic endosomes^55^ and its vesicular localization is controlled by Rab8a.^35^ Therefore, we examined the endosomal distribution of Ptc1 in RNF126-depleted cells. In *control* siRNA-treated cells, the majority of Ptc1 was detected in cytoplasmic vesicular structures (Figures 6C–a), as reported previously.^35^^,^^55^ In contrast, Ptc1 was present mostly in congregated perinuclear vesicular compartments and reduced cytoplasmic endosomal structures in RNF126-defective cells (Figures 6C–b and S4). Double depletion of two related E3 ligases (RNF126 and RNF115) also altered Ptc1 distribution (Figures 6C–c). Note that the expression level of Ptc1-Flag protein was not affected by *RNF126* siRNA (Figure 6D), and no overt difference in the distribution of early endosome marker EEA1 was observed in RNF126-depleted cells (Figure S4). These results indicate that endogenous RNF126-family ubiquitin ligases play a role in controlling the localization of Rab8a client membrane proteins.

### Pre-emptive quality control machinery is necessary for primary cilium formation

The primary cilium is a microtubule-based sensory organelle that protrudes from the surface of almost all vertebrate cells.^56^ The formation of primary cilia (i.e., ciliogenesis) requires polarized vesicular trafficking events.^56^^,^^57^ Importantly, Rab10 depletion in *Rab8a/b* double knockout cells has been reported to lead to a significant reduction of ciliation,^49^ suggesting that these paralogs have a redundant but critical role in ciliogenesis.^31^^,^^32^^,^^49^^,^^58^^,^^59^ Given the present finding that the pre-emptive quality control system is essential for eliminating GDP-bound Rab8 and Rab10, this quality control machinery may be necessary for ciliogenesis. We therefore, tested this hypothesis by depleting several components of the pre-emptive quality control machinery.

**Figure 7:**
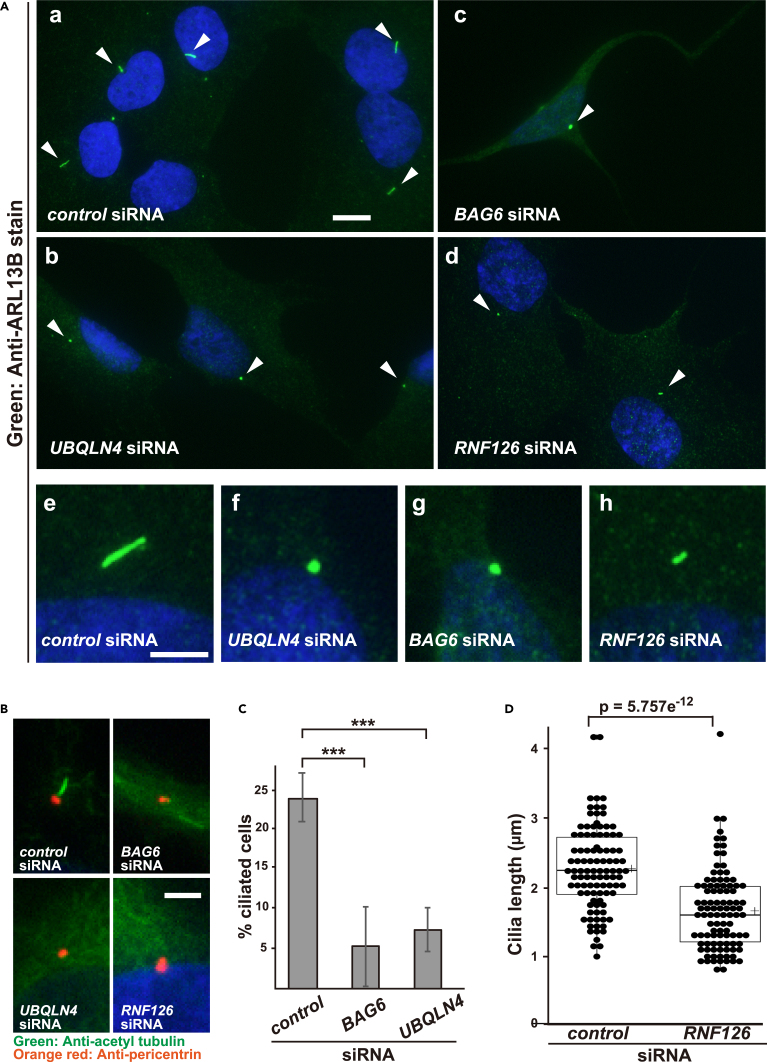
Depletions of pre-emptive quality control machinery components prevent ciliogenesis (A) Primary cilium formation in hTERT-RPE1 cells treated with *control* siRNA (a), *UBQLN4* siRNA (b), *BAG6* siRNA (c), and *RNF126* siRNA (d). At 72 h after transfection with the indicated siRNA duplexes (5 nM each), hTERT-RPE1 cells were serum-starved for 24 h to trigger ciliogenesis, and the distribution of ARL13B, a marker for primary cilia, was observed (shown in green). Nuclei were stained with Hoechst 33258 (shown in blue). Cells treated with *control* siRNA grew primary cilia (a, rods in green channel, indicated as arrowheads), whereas those treated with knockdown of *UBQLN4* (b), *BAG6* (c), and *RNF126* (d) failed to grow cilia. Scale bar: 10 μm. Magnified independent images of primary cilia (e–h). Scale bar: 3 μm. Note that all images presented in this figure were acquired in an identical set of experiments, and the exposure times of all photographs in this figure were the same. (B) Serum-starved hTERT-RPE1 cells were immunostained with acetylated α-tubulin (a cilial axoneme marker, green) and pericentrin (a basal body marker, red) in siRNA treatments for pre-emptive quality machinery components. Control cells grew primary cilia (rods in the green channel), whereas the cells with BAG6, UBQLN4, and RNF126 depletions failed to grow cilial axonemes on the basal bodies. Nuclei were stained with Hoechst 33258 (shown in blue). Scale bar: 3 μm. See also Figure S5. (C) The percentage of ciliated cells after depletion of pre-emptive quality control machinery components. Cilia were counted by scoring ARL13B-positive structures greater than 1 μm in length with at least three biologically independent replicates. All analyzed experiments used biological replicates to compute statistical significance. Data are presented as means ± SD, and were analyzed using Dunnett’s test. **∗∗**∗p < 0.001. (D) Cilia in *RNF126* siRNA-treated cells had decreased length. The Mann-Whitney *U* test was used for statistical analysis. Center lines in the boxplots show the medians; crosses represent sample means; n = 100 for each treatment group; p = 5.757e^−12^.

To examine the possible participation of the pre-emptive quality control machinery in serum starvation-induced ciliogenesis, we depleted the gene products of this machinery in hTERT-RPE1 cells, a human immortalized retinal pigment epithelial cell line. Control cells grew primary cilia (Figures 7A–a, and e; rods in the green channel as a cilia marker of ARL13B-positive structures, indicated by white arrowheads), while UBQLN4 depletion drastically impaired ciliogenesis (Figures 7A-b, and f). Similar observations were made for the depletion of BAG6 (Figures 7A–c, and g) and RNF126 (Figures 7A–d, and h). We measured the percentage of ciliated cells by scoring ARL13B-positive structures greater than 1 μm in length. We found that 23.6% of cells (255/1082 cells) were ciliated in the presence of *control* siRNA, whereas only 6.1% of *BAG6* siRNA-treated cells (21/344 cells) were ciliated (Figure 7C). Depletion of *UBQLN4* by siRNA also resulted in shorter primary cilia (7.1%, 37/522 cells). Although *RNF126* knockdown did not significantly reduce the rate of ciliated cells having cilia longer than 1 μm, we found that the average length of primary cilia was significantly shorter in *RNF126* siRNA-treated cells than in control cells (Figure 7D; average length: 1.6 μm in RNF126-depleted cells, 2.3 μm in control cells).

The cilium is assembled from the distal end of the mother centriole or basal body, which serves to nucleate growth of the axoneme, which is a microtubule doublet. To examine the formation and position of the axoneme on the basal body, we double-stained serum-starved RPE1 cells with antibodies against anti-acetyltubulin (a cilia axoneme marker) and anti-pericentrin (a basal body marker). After 48 h of serum starvation, the mother centrioles were associated with the ciliary pockets with extended axonemal shafts (Figures 7B and S5A). Depletion experiments of pre-emptive quality control components, including UBQLN4, RNF126, and BAG6, confirmed a reduction in the number of elongated axonemes on the normal basal body, as determined by acetylated tubulin and pericentrin staining (Figures 7B and S5A–S5D). These observations further support the critical function of these proteins as degrading machinery for ciliogenesis. We confirmed that endogenous Rab8a protein in RPE1 cells can be physically recognized by BAG6 and that their interaction was stimulated by both MG-132 treatment and *RABIN8* siRNA (Figure S5E). These observations suggest that the proteasome is responsible for the elimination of endogenous GDP-bound Rab8a in the RPE1 cells.

Cliogenesis and cell division are mutually exclusive events, and primary cilia are known to be formed specifically during the G0/G1 phase. To examine whether the phenotypes observed in this study are not the results of secondary cell-cycle defects, we performed flow cytometric analysis upon depletion of the pre-emptive quality control machinery. Knockdown of UBQLN4 or RNF126 did not lead to any decrease in the G0/G1 populations of serum-depleted RPE1 cells (95.5% and 96.7% G0/G1 cells, respectively, Figure S6) compared with the control (95.4% G0/G1 cells). Although BAG6 depletion showed a slight reduction in the G0/G1 population (89.8% G0/G1 cells in BAG6 depletion) as previously reported (94% of G0/G1 cells in control cells, 87% in BAG6 depleted cells),^60^ the observed effect was very small. We concluded that the reduced G0/G1-phase population was not a major cause of the overt defects in cilia formation in UBQLN4-, RNF126-, and BAG6-defective cells. Taken together, these findings suggest that the pre-emptive quality control machinery have an indispensable role in ciliogenesis.

## Discussion

The Rab family of small GTPases has long been thought to be stable proteins; indeed, the half-lives of Rab8a and Rab10 in their GTP-bound form are very long. In contrast to this generally believed notion, our study suggested that the cryptic portion of Rab8, that is a GDP-bound form, is extremely unstable. The instability of GDP-bound Rab8a seems not to be caused by unexpected structural defects resulting from the T22N mutation, given that the amount of wild-type Rab8a that co-precipitated with UBQLN4 under GEF suppression was also augmented after proteasomal inhibition (Figure 2F). Similarly, polyubiquitination of wild-type Rab8a was stimulated following GEF depletion and proteasomal inhibition (Figures 3E and 3F). Although we can’t exclude the possibility that T22N mutation may alter the protein stability in ways unrelated to the nucleotide-bound state, we think this is largely unlikely. Wild-type Rab8a looks highly stable as a whole because the equilibrium between GTP- and GDP-binding to Rab8a is largely in favor of GTP.

The chaperone protein BAG6 plays a role in the instability of GDP-bound Rab8 subfamily proteins. BAG6 is a multifunctional protein that participates in diverse processes, such as protein biogenesis and degradation, with many distinct regulatory subunits dedicated to these respective processes. For example, BAG6 associates with the transmembrane recognition complex (TRC) to facilitate the biogenesis of tail-anchored membrane proteins.^42^^,^^61^^,^^62^ It is also a component of the pre-emptive protein quality control machinery.^38^^,^^45^^,^^47^ Therefore, distinguishing the machinery required for BAG6-dependent Rab8a regulation is important. In this study, we provide the first evidence that pre-emptive quality control components, including RNF126 and UBQLN4, are responsible for the instability of GDP-bound Rab8 subfamily proteins (Figures 2B, 5A, 5C, and 5D). The pre-emptive quality control system is known to selectively target aggregation-prone hydrophobic clients to BAG6-associated ubiquitin ligases such as RNF126 and the ubiquitin receptor UBQLN4.^38^^,^^39^^,^^45^^,^^47^^,^^63^^,^^64^ Because Rab8a exposes its hydrophobic stretch in a GDP-bound form-specific manner^51^ and it has been reported that GDP-bound Rab8a is aggregation-prone in the cytoplasm,^29^ GDP-bound Rab8 can be a preferential target for these components of the ubiquitination machinery.

Since Rab10 protein is post-translationally modified by highly lipophilic geranylgeranyl groups (consisting of 20-carbon isoprenoid groups) at the C-terminal Cys199 and Cys200 residues, we suspected that these hydrophobicities might contribute to its instability. To examine whether these geranylgeranylations are essential for the association of Rab10-T23N with BAG6, we mutated cysteine residues to serine (designated C199S/C200S mutant Rab10, Figure S7A). We found that C199S/C200S mutations of Rab10 did not reduce its BAG6 binding as the GDP-bound cytosolic form (Figure S7B). In accordance with this, C199S/C200S mutant Rab10 remains highly unstable (Figure S7C), suggesting that C-terminal geranylgeranyl groups are not responsible for rapid degradation of GDP-bound Rab10. These observations are consistent with our previous report that the exposed hydrophobicity resulting from isoprenylation of the C-terminus is not involved in the instability of GDP-bound Rab8a.^35^ This view is further supported by our observation that the overexpression of GDP dissociation inhibitor 2 (GDI2), a known cytoplasmic chaperone dedicated for isoprenylated Rab proteins in its GDP-bound form (Figure S7A), did not stabilize Rab8a-T22N (Figure S7D) or Rab10-T23N (Figure S7E) in HeLa cells. Therefore, a shortage of GDI is not sufficient to explain the observed instability of GDP-bound Rab8a subfamily proteins.

**Figure 8:**
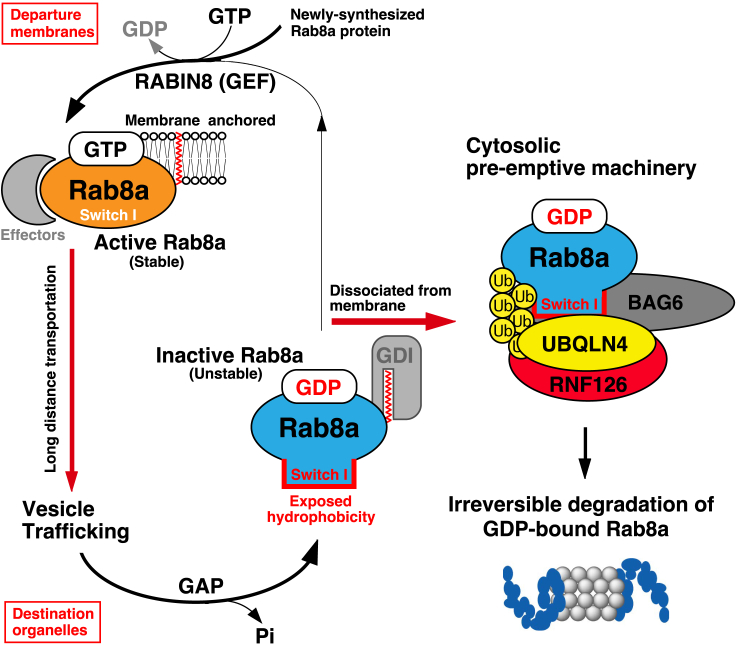
Pre-emptive quality control machinery targets GDP-bound Rab8a/10 for ubiquitin-dependent degradation GDP-bound Rab8a/10 released from the destination membranes is thought to return to its departure organelles where it is reactivated by specific GEFs. In this study, we show that a non-negligible portion of GDP-bound Rab8a/10 is targeted for degradation by the pre-emptive quality control machinery. The exposed hydrophobic region of GDP-bound Rab8a in Switch I is critical for its ubiquitin-mediated degradation, thereby preventing the excessive accumulation of inactive Rab species during GDP-GTP cycling. The association of pre-emptive quality control machinery components with wild-type Rab8a was greatly enhanced under RABIN8 (GEF)-suppressed conditions. Furthermore, depletions of BAG6, UBQLN4, and RNF126 resulted in the stabilization of GDP-bound Rab8a/10, thereby preventing ciliogenesis. These results suggest that the protein degradation machinery plays a critical role in maintaining the integrity of membrane trafficking events by limiting the excessive accumulation of GDP-bound forms of Rab8a/10 small GTPases. See also Figures S7 and S8.

Rab8-family proteins are responsible for vesicular trafficking between cytoplasmic organelles. Unlike Ras (Figure S8), GTP hydrolysis and the subsequent retrieval of Rab8a from membranes occur at locations far from the departure organelles. It remains unclear how GDP-bound Rab8a generated at the destination membranes returns to its departure organelles through long-distance transportation. We suggest that most, if not all, GDP-bound Rab8a and Rab10 must be targeted immediately for degradation after cytoplasmic release from the destination membranes (Figure 8). In relation to this, it has been reported that the accumulation of GDP-bound Rab proteins in the cytoplasm is detrimental to intracellular membrane trafficking.^23^^,^^24^^,^^25^^,^^26^^,^^27^^,^^28^^,^^29^^,^^30^ The inadequate elimination of GDP-bound Rab8a might cause the unregulated accumulation of aggregation-prone Rab8a (Figures 6A and 6B), which might impair vesicular trafficking.^17^^,^^31^^,^^32^^,^^65^ Therefore, pre-emptive quality control-mediated degradation of Rab8-subfamily proteins during GDP/GTP cycling are necessary for selectively limiting the excessive accumulation of Rab8 in its GDP-bound form to maintain the integrity of vesicular trafficking (Figure 8).

In this context, it is interesting to recall previous reports that RNF126 plays a role in some membrane trafficking events via an unknown mechanism. For example, RNF126 is involved in the cytoplasmic incorporation of cell surface receptors^66^ and plays a role in the efficiency of endosomal sorting.^67^ Additionally, the golgi apparatus is mildly disrupted in RNF126-depleted cells,^67^ similar to the case in BAG6 depletion.^35^ Although the substrates of the RNF126 ubiquitin ligase in this context were poorly investigated, the data in the present study suggest that this ubiquitin ligase is necessary for preventing the accumulation of GDP-bound Rab family small GTPases.

Recently, it was reported that RNF115, an RNF126 paralog, interacts with Rab1A and Rab13 predominantly in their GDP-bound forms, thereby inducing K11-linked polyubiquitin modifications.^68^ RNF115 modulates the vesicular trafficking of toll-like receptor from the ER/golgi apparatus to the cell surface.^68^ Since residues identified as ubiquitination sites of Rab1A (Lys49 and Lys61) and Rab13 (Lys46 and Lys58) were conserved in multiple Rab proteins, including Rab8a and Rab10,^68^ it is conceivable that Rab8-family proteins were also polyubiquitinated on these residues. Although proteasomal degradation of Rab1A and Rab13 has not been reported, our preliminary study suggests that GDP-bound Rab13 is highly unstable in HeLa cells, such as in the case of Rab8a-T22N and Rab10-T23N mutants.

A notable finding in this study was that primary cilia formation was disrupted in cells with depleted components of the pre-emptive quality control machinery (Figure 7 and S5). Depletion of these components also disrupted the intracellular localization of the sonic hedgehog receptor Ptc1 (Figure 6C). The sonic hedgehog morphogen requires Rab8 subfamily proteins for transduction of its intermediate signaling at the tip of the primary cilium.^69^ Accordingly, it has been reported that the forced accumulation of GDP-locked variants of Rab8a or depletion of RABIN*8* abolishes ciliogenesis in hTERT-RPE1 cells.^31^^,^^32^ In addition, the Exocyst-Rab8-RABIN8 complex has a crucial role in the docking of exocytic vesicles to the plasma membrane and is necessary for primary ciliogenesis.^59^^,^^70^ Given that defects in primary cilia are associated with the defective transduction of sonic hedgehog signals^71^ and with a large class of inherited disorders collectively known as ciliopathies,^56^^,^^71^^,^^72^ the pre-emptive quality control system might possess huge importance in the pathomechanism of ciliary function-associated disorders. This should be explored further in future study.

We recently found that BAG6 is necessary for stress fiber formation via supporting RhoA stability.^73^ Since blocking stress fiber was reported to facilitate ciliogenesis and results in abnormally long cilia,^74^ observed defects in cilial elongation as a result of BAG6 depletion are consequence of the predominant suppression of cilia over the effects of defective stress fiber formation. In addition, neither UBQLN4 nor RNF126 is involved in the formation of stress fiber,^73^ suggesting that the observed phenotypes of UBQLN4 and RNF126 depletion in ciliogenesis are independent of stress fiber formation.

An interesting issue that remains to be elucidated is whether the pre-emptive quality control machinery is responsible for other Rab family proteins. The human genome encodes more than 60 *Rab* genes.^9^^,^^75^ The instability of GDP-bound Rab proteins seems to be subfamily specific (Figures 1A–1D) and partially correlates with their affinity for BAG6. Many mammalian Rab family proteins show high binding affinity with BAG6,^35^ thus, these BAG6-phile Rab proteins might be potential clients for pre-emptive quality control in a nucleotide-specific manner. Therefore, the effects of defective pre-emptive quality control on membrane trafficking might not solely rely on the dysfunction of Rab8a and Rab10. RNF126 has also been reported to play a role in retrograde sorting of the cation-independent mannose 6-phosphate receptor.^66^ Considering that vesicular trafficking of the mannose 6-phosphate receptor is mediated by Rab9a and that Rab9a has the highest affinity for BAG6,^35^ Rab9a might be an excellent candidate client protein for pre-emptive quality control. We are investigating this possibility in parallel studies.

In summary, we found evidence that the pre-emptive quality control machinery is essential for proper membrane trafficking via the irreversible degradation of Rab8 subfamily proteins in a nucleotide-specific manner (Figure 8). This finding implies that the simple nucleotide exchange cycle (as shown in the case of Ras, Figure S8) might not be sufficient to explain the regulation of Rab8 subfamily small GTPases, contrary to the currently accepted paradigm. We speculate that the cytoplasmic transport of GDP-bound Rab8a back to the departure organelle may be, at least in part, dispensable. Instead, excessive amounts of GDP-bound Rab8 subfamily proteins must be degraded immediately (Figure 8). The importance of the degradation of other Rab family small GTPases should be explored further to determine the fate of these mobile proteins after GTP hydrolysis at the end of their journey.

### Limitations of the study

This study showed Rab8a and Rab10 in their GDP-bound forms are highly unstable, whereas Rab7a is stable in HeLa cells. The instabilities of other GDP-bound Rab-family proteins remain an important area for future investigation. Most of the experiments in this study were performed in cells ectopically expressing the Rab GTPases. Although, we demonstrated that co-precipitation of endogenous Rab8a with BAG6-probe was stimulated by GEF suppression and MG-132 treatment, direct estimation of the endogenous level of GDP-bound Rab8a should be necessary. Finally, a detailed mechanistic understanding of how RNF126 cooperates with BAG6 to promote polyubiquitination of GDP-bound Rab8a will require biochemical reconstitution from purified components. Such studies will be crucial for elucidating the mechanism by which pre-emptive protein quality control machinery selectively regulates specific species of Rab-family proteins in a nucleotide-dependent manner.

## STAR★Methods

### Key resources table

**Table:** 

REAGENT or RESOURCE	SOURCE	IDENTIFIER
**Antibodies**		

anti-BAG6 rabbit polyclonal	Minami et al.^37^	RRID: AB_2934122
anti-Rab8a mouse monoclonal	BD Biosciences	Cat# BD610844; RRID: AB_398164
anti-polyubiquitin FK2 mouse monoclonal	MBL	Cat# D058-3; RRID: AB_592937
anti-polyubiquitin FK2 mouse monoclonal	Nippon Bio-Test Laboratories Inc.	Cat# 0918-2; RRID: AB_2934121
anti-RNF126 rabbit polyclonal	Abcam	Cat# ab183102; RRID: AB_2934116
anti-RNF115 rabbit monoclonal	Abcam	Cat# ab187642; RRID: AB_2934119
anti-UBQLN4 (anti-A1Up) mouse monoclonal	Santa Cruz Biotechnology, Inc.	Cat# sc-136145; RRID: AB_2934119
anti-Flag M2 mouse monoclonal	Sigma-Aldrich	Cat# F1804; RRID: AB_262044
anti-Flag M2 affinity gel	Sigma-Aldrich	Cat# A2220; RRID: AB_10063035
anti-Flag rabbit polyclonal	Sigma-Aldrich	Cat# F7425; RRID: AB_439687
anti-T7-tag monoclonal	Novagen	Cat# 69522-3; RRID: AB_11211744
anti-T7 tag antibody agarose	Novagen	Cat# 69026-3; RRID: AB_10947861
anti-β-actin rabbit polyclonal	Sigma-Aldrich	Cat# A2066; RRID: AB_476693
anti-calnexin polyclonal	Sigma-Aldrich	Cat# C4731; RRID: AB_476845
anti-α-tubulin (TU-02) mouse monoclonal	Santa Cruz Biotechnology, Inc.	Cat# sc-8035; RRID: AB_628408
anti-α-tubulin (DM1A) mouse monoclonal	Sigma-Aldrich	Cat# T9026; RRID: AB_477593
anti-RABIN8/RAB3IP rabbit polyclonal	Proteintech	Cat# 12321-1-AP; RRID: AB_2177510
anti-ARL13B mouse monoclonal	Neuromab	Cat# 75–287; RRID: AB_2341543
anti-acetylated α-tubulin mouse monoclonal 6-11B-1	Abcam	Cat# ab24610; RRID: AB_448182
anti-pericentrin rabbit polyclonal	Abcam	Cat# ab4448; RRID: AB_304461
anti-EEA1 rabbit polyclonal	Cell signaling	Cat# 2411; RRID: AB_2096814
Alexa Fluor^R^488 goat anti-mouse immunoglobulin	Thermo Fisher Scientific	Cat# A11001; RRID: AB_2534069
Alexa Fluor^R^594 goat anti-rabbit immunoglobulin	Thermo Fisher Scientific	Cat# A11037; RRID: AB_2534095
Horseradish peroxidase-conjugated antibody against mouse immunoglobulin	GE Healthcare	Cat# NA931; RRID: AB_772210
Horseradish peroxidase-conjugated antibody against rabbit immunoglobulin	GE Healthcare	Cat# NA934; RRID: AB_772206

**Chemicals, peptides, and recombinant proteins**		

MG-132 (Z-Leu-Leu-Leu-CHO)	Peptide institute	Cat# 3175-v; CAS 133407-82-6
Bortezomib	Fujifilm-Wako	Cat# 021–18901; CAS 179324-69-7
TAK-243 (MLN7243)	Selleck	Cat# S8341; CAS 9005-64-5
Cycloheximide	Fujifilm-Wako	Cat# 033–20993; CAS 66-81-9
N-Ethylmaleimide	Fujifilm-Wako	Cat# 056–02062; CAS 128-53-0
Protease inhibitor cocktail	Fujifilm-Wako	Cat# 161-26023
S-protein agarose	Novagen	Cat# 69704
IGEPAL (Nonidet P-40)	Sigma-Aldrich	Cat# I3021; CAS 9002-93-1
Polyoxyethylene (10) octylphenyl ether (Triton X-100)	Fujifilm-Wako	Cat# 162–24755; CAS 9002-93-1
Paraformaldehyde	Nacarai tesque	Cat# 26126-25; CAS 30525-89-4
Hoechst 33258 solution	Dojindo	Cat# H341; CAS 23491-45-4
Dulbecco’s modified Eagle medium–nutrient mixture F-12 (DMEM/F12) medium	ATCC	Cat# 30-2006
Dulbecco’s Modified Eagle Medium (DMEM: High glucose) with L-Glitamine, Phenol red and Sodium pyruvate	Fujifilm-Wako	Cat# 043-30085
Opti-MEM™ I Reduced Serum Medium	Thermo Fisher Scientific	Cat# 31985070
0.25 w/v% Trypsin-1 mmol/L EDTA. 4Na Solution with Phenol red	Fujifilm-Wako	Cat# 201-16945
Fetal Bovine Serum	Invitrogen	Cat# 10437028
Fetal Bovine Serum	Hyclone	Cat# SH30396.03
Fetal Bovine Serum	Nichirei	Cat# 175012
Propidium iodide	Sigma-Aldrich	Cat# P4170; CAS 25535-16-4
RNase A	NIPPON GENE	Cat# 318-06391
Dimethyl sulfoxide (DMSO)	Fujifilm-Wako	Cat# 043–29355; CAS 67-68-5
WIDE-VIEW Prestained Protein Size Marker III	Fujifilm-Wako	Cat# 234-02464
Immobilon-P transfer membrane	Merck Millipore	Cat# IPVH00010
Fluoromount	Diagnostic biosystem	Cat# K024
HilyMax	Dojindo	Cat# H357
PEI MAX - Transfection Grade Linear Polyethylenimine Hydrochloride (MW 40,000)	Polysciences, Inc	Cat# 24765-1; CAS 49553-93-7
Lipofectamine RNAiMAX	Thermo Fisher Scientific	Cat# 13778150
Tween 20	Sigma-Aldrich	Cat #P1379; CAS 9005-64-5

**Critical commercial assays**		

Can Get Signal® Immunoreaction Enhancer Solution 1 & 2	TOYOBO	Cat# NKB-101; CAS 9000-71-9
Clarity Wester ECL Substrate	Bio-Rad	Cat# 1705061
Favorprep Plasmid DNA Extraction Midi Kit	Favorgen	Cat# FAPDE002-1
FastGene Plasmid Mini Kit	NIPPON Genetics	Cat# FG-90502

**Experimental models: Cell lines**		

Human: hTERT-RPE1, female-derived cell line	ATCC	Cat# CRL-4000
Human: HEK293T	RIKEN BRC	BRC# RCB2202
Human: HeLa, female-derived cell line	RIKEN BRC	BRC No# RCB0007

**Oligonucleotides**		

siRNA targeting sequence: *RABIN8* 5′-CAUUGAAGACACUUGUAUUTT-3′	Takahashi et al.^35^	N/A
siRNA targeting sequence: *BAG6* #1 5′-UUUCUCCAAGAGCAGUUUATT-3′	Minami et al.^37^	N/A
siRNA targeting sequence: *BAG6* #6 5′-ACCGGAAUGCCAACAGCUATT-3′	Miyauchi et al.^73^	N/A
siRNA targeting sequence: *RNF126* #1 5′- CAUCCCGGACGGUACUUCUTT-3′	Rodrigo-Brenni et al.^45^	N/A
siRNA targeting sequence: *RNF126* #3 5′- CUUUCGGCAUCUUCGAUGATT-3′	This paper	N/A
siRNA targeting sequence: *RNF115* #2 5′- CUUGCAAUCACUUCUUUCATT-3′	This paper	N/A
siRNA targeting sequence: *UBQLN4* #3 5′-CUCUUCAGAUGCUGGCAGUTT-3′	Suzuki and Kawahara,^47^	N/A
AllStars Negative Control siRNA	QIAGEN	Cat# 1027281

**Software and algorithms**		

ImageJ ver. 1.53s	NIH	https://imagej.nih.gov/ij/
R statistical software (4.1.3)	R Core Team 2022	https://www.r-project.org/
FCS Express 7	*De Novo* Software	ver. 7
Excel 2016	Microsoft	https://www.office.com/
Photoshop	Adobe	21.1.0
Illustrator	Adobe	24.1

### Resource availability

#### Lead contact

Further information and requests for resources and reagents should be directed to and will be fulfilled by the lead contact, Hiroyuki Kawahara (hkawa@tmu.ac.jp).

#### Materials availability

Unique reagents generated in this study are available from the lead contact with a Materials Transfer Agreement.

### Experimental model and subject details

HeLa cells were purchased from RIKEN cell bank, and was cultured in Dulbecco’s modified Eagle’s medium (Sigma Chemical Co., St Louis, MO) supplemented with 10% heat-inactivated calf serum at 37 °C under a 5% CO_2_ atmosphere. Human telomerase reverse transcriptase retinal pigment epithelium 1 (hTERT-RPE1) cells were purchased from American Type Culture Collection (ATCC), and were cultured according to the manual provided. Briefly, cells were cultured at 37°C under 5% CO_2_ in Dulbecco’s modified Eagle medium–nutrient mixture F-12 (DMEM/F12) medium (ATCC) supplemented with 10% fetal bovine serum (FBS).

### Method details

#### Plasmid construction

Full-length cDNAs for UBQLN4, RNF126, RNF115, BAG6, Ptc1, GDI2 and Rab-family proteins were amplified by PCR from cDNA library derived from HEK293 cells. The PCR fragments were cloned into the pCI-neo expression vector (Promega, Madison, WI) for expression in cultured cells. It should be noted that expression vectors encode three repeats of a Flag-, T7-, or single S-tags at the N-terminus of their products. The truncated and mutated versions of the products were constructed by PCR. These expression vectors were used for experiments after verification of the sequence of inserted DNA.

#### Cell culture and transfection

HeLa cell was cultured in Dulbecco’s modified Eagle’s medium (Sigma Chemical Co.) supplemented with 10% heat-inactivated calf serum at 37 °C under a 5% CO_2_ atmosphere. Transfection in HeLa cells with pCI-neo expression vectors was performed by using PEI MAX (Polysciences, Inc, PA, USA) or HilyMax (Dojindo, Kyoto, Japan) according to the protocols supplied by the manufacturers. At 24 h after plasmid transfection, the cells were harvested and subjected to immunological analysis, unless otherwise noted.

Human telomerase reverse transcriptase retinal pigment epithelium 1 (hTERT-RPE1) cells were purchased from American Type Culture Collection (ATCC), and were cultured according to the manual provided. Briefly, cells were cultured at 37°C under 5% CO_2_ in Dulbecco’s modified Eagle medium–nutrient mixture F-12 (DMEM/F12) medium (ATCC) supplemented with 10% fetal bovine serum (FBS).

#### RNA interference

*UBQLN4* depletion in human cells was performed with duplex siRNAs covering the targeted sequences;

5′-CUCUUCAGAUGCUGGCAGUTT-3′ (*UBQLN4* siRNA#3)

*RABIN8* depletion in human cells was performed with duplex siRNAs covering the targeted sequences;

5′-CAUUGAAGACACUUGUAUUTT-3′

*BAG6* depletion in human cells was performed with two independent duplex siRNAs covering the targeted sequences;

5′-UUUCUCCAAGAGCAGUUUATT-3′ (*BAG6* #1)

5′-ACCGGAAUGCCAACAGCUATT-3′ (*BAG6* #6)

*RNF126* and *RNF115* depletions in human cells were performed with duplex siRNAs covering the targeted sequences;

5′- CAUCCCGGACGGUACUUCUTT-3′ (*RNF126* siRNA#1)

5′- CUUUCGGCAUCUUCGAUGATT-3′ (*RNF126* siRNA#3)

5′- CUUGCAAUCACUUCUUUCATT-3′ (*RNF115* siRNA#2).

*RNF126* siRNA#3 was used for supporting the reproducibility of Figures 5A and 5B.

As a negative control for siRNA experiments, AllStars Negative Control siRNA (QIAGEN 1027281) was used unless otherwise noted. Transfections with duplex siRNA were performed using Lipofectamine RNAiMAX (Thermo Fisher Scientific), according to the protocols provided by the manufacturer. The efficacy of each siRNA was verified by immunoblot with their specific antibodies listed in the latter section.

#### Immunological analysis

For immunoprecipitation analysis, HeLa cells were washed with phosphate-buffered saline (PBS) and lysed with immunoprecipitation (IP) buffer containing 25 mM Tris-HCl pH7.5, 150 mM NaCl, 5 mM EDTA, 10% glycerol, 1% Nonidet P-40, 20 μM MG-132. The lysates were pipetted, centrifuged at 20,630 x g for 10–20 min at 4 °C, and mixed with 4–10 μL of anti-Flag M2 affinity gel (Sigma) or anti-T7 tag antibody agarose (Novagen) for 5 min to 2 h at 4°C. After the beads had been washed five times with the IP buffer, the immuno-complexes were eluted with SDS sample buffer.

For Western blot analyses, whole cell lysates and the immunoprecipitates were subjected to SDS-PAGE and transferred onto polyvinylidene difluoride transfer membrane (Merck-Millipore, Cork, Ireland). The membranes were then immunoblotted with specific antibodies as indicated and then incubated with horseradish peroxidase-conjugated antibody against mouse or rabbit immunoglobulin (GE Healthcare), followed by detection with ECL Western blotting detection reagents (GE Healthcare), Clarity Western ECL substrate (Bio-RAD).

For hot lysis analysis, HeLa cells were washed twice with PBS and lysed with Hot lysis buffer containing 1% SDS, 50 mM Tris-HCl pH7.5, 150 mM NaCl, 5 mM EDTA, 25 μM MG-132, 10 mM N-ethylmaleimide and protease inhibitor cocktail. The lysates were heat-treated at 90°C for 15 min and sonicated. After the lysates were centrifuged at 20,630 x g for 20 min at room temperature, the supernatants were diluted for 4-fold with buffer A (containing 1% Triton X-100, 50 mM Tris-HCl pH7.5 and 150 mM NaCl). Diluted extracts were mixed with 5 μL anti-Flag M2 affinity gel beads (Sigma) for 10 min at 4°C. After the beads had been washed five times with buffer A, the bounded immuno-complexes were eluted by SDS sample buffer and subsequently subjected to Western blot analyses. Note that independent experiments were repeated at least three times to ensure the reproducibility of the data. Information about the primary and secondary antibodies and the dilutions are provided in key resources table.

#### Immunocytochemical observations of Ptc1

For the immunostaining of Ptc1 in HeLa cells, we used pCI-neo-based modified expression plasmid with largely compromised promoter activity (by deleting promoter region partially) to keep the expression of Flag-tagged Ptc1 protein (Ptc1-Flag) at nearly physiological levels.^35^ This was due to preclude the possibility of inappropriate aggregation of this polytopic TMD protein in the cytosol. Transfected HeLa cells were grown on micro coverglass (Matsunami, Osaka, Japan), fixed by incubating in 4% paraformaldehyde for 10 min at room temperature, and permeabilized with 0.1% Triton X-100 for 3 min at room temperature. All cells after fixation and permeabilization were blocked with 3% calf serum solution in PBS for 30 min at room temperature, reacted with anti-Flag M2 primary antibody at 4°C for overnight, and were subsequently reacted with secondary antibody, Alexa Fluor^R^488 goat anti-mouse IgG. To observe the nucleus, cells were treated with 2.5 μg/mL Hoechst 33258. Immunofluorescent images were obtained by BIOREVO BZ9000 fluorescence microscope (Keyence, Osaka, Japan).

#### Primary cilia observation

One day after plating of hTERT-RPE1 cells, siRNAs (5 nM) were transfected into the cells by using Lipofectamine RNAi MAX (Thermo Fisher Scientific) according to the manufacturer’s instructions, and cultured for 72 h in complete medium to approximately 60–70% confluence. To induce ciliogenesis, the complete medium was replaced with serum-free medium, and the cells were cultured for a further 24 h. For immunocytochemical observations of primary cilia, hTERT-RPE1 cells were fixed in 4% paraformaldehyde in PBS for 15 min at room temperature, and after two washes with PBS, the cells were permeabilized in ice-cold methanol for 5 min. Then, cells were blocked in 5% FBS-PBS for 30 min at room temperature. Cilia were analyzed by immunostaining of endogenous ARL13B, a marker for primary cilia, with anti-ARL13B antibody or anti-acetylated α-tubulin as primary antibodies, and were subsequently reacted with a secondary antibody, Alexa Fluor^R^488 goat anti-mouse IgG. To evaluate the length and frequency of primary cilia, ARL13B-positive immunosignals were measured using ImageJ software under the BZ-9000 fluorescence microscope (Keyence). Data for at least 100 cilia per treatment were obtained from at least three independent biological replicates, and the values are presented as means ± S.D.

#### Flow cytometry for cell cycle analysis

For quantification of cell cycle stage distribution, the hTERT-RPE1 cells were washed twice with PBS, and harvested by trypsinization from culture plates. The cells were re-suspended in 500 μL PBS, and 1 mL of ethanol was subsequently added to the cell suspension. After incubation at 4°C for at least 1 h, the cells were washed 3 times with PBS. The cells were suspended again in PBS and incubated at 4°C for 30 min. After incubation, RPE1 cells were centrifuged and the supernatant was removed. Next, 0.5 mL of 250 U/mL RNase A in PBS was added to cell, and the cells were incubated at room temperature for 20 min. After incubation, 50 μg/mL propidium iodide (PI) was added. The cell cycle profile was analyzed using a flow cytometer (model BD Accuri C6, BD Bioscience) by 488 nm excitation.^76^ FCS files from cell cycle assay were extracted and analyzed using FCS Express 7.

### Quantification and statistical analysis

Statistical analysis was performed using R (4.1.3) or Microsoft Excel 2016. Statistical details of experiments are stated in the legends of figures displaying the respective data, including the statistical tests used, the number of replicates and number of investigated cells, and measures of precision. For two-sample comparisons, students' t-test or the Mann-Whitney U test was used. For multiple comparisons, statistical significance was tested by one-way ANOVA and Dunnett’s test. p value <0.05 was considered statistically significant (p < 0.05∗, 0.01∗∗, 0.001∗∗∗). Note that independent experiments were repeated at least three times to ensure the reproducibility of the data.

## Data Availability

• All data reported in this paper will be shared by the lead contact upon request. • This paper does not report original code. • Any additional information required to reanalyze the data reported in this paper is available from the lead contact upon request.

## References

[1] Bos J.L., Rehmann H., Wittinghofer A. (2007). GEFs and GAPs: critical elements in the control of small G proteins. Cell.

[2] Hennig A., Markwart R., Esparza-Franco M.A., Ladds G., Rubio I. (2015). Ras activation revisited: role of GEF and GAP systems. Biol. Chem..

[3] Buday L., Downward J. (1993). Epidermal growth factor regulates p21ras through the formation of a complex of receptor, Grb2 adapter protein, and Sos nucleotide exchange factor. Cell.

[4] Li N., Batzer A., Daly R., Yajnik V., Skolnik E., Chardin P., Bar-Sagi D., Margolis B., Schlessinger J. (1993). Guanine-nucleotide-releasing factor hSos1 binds to Grb2 and links receptor tyrosine kinases to Ras signalling. Nature.

[5] Aronheim A., Engelberg D., Li N., al-Alawi N., Schlessinger J., Karin M. (1994). Membrane targeting of the nucleotide exchange factor Sos is sufficient for activating the Ras signaling pathway. Cell.

[6] Fukuda M. (2008). Regulation of secretory vesicle traffic by Rab small GTPases. Cell. Mol. Life Sci..

[7] Stenmark H. (2009). Rab GTPases as coordinators of vesicle traffic. Nat. Rev. Mol. Cell Biol..

[8] Hutagalung A.H., Novick P.J. (2011). Role of Rab GTPases in membrane traffic and cell physiology. Physiol. Rev..

[9] Zhen Y., Stenmark H. (2015). Cellular functions of Rab GTPases at a glance. J. Cell Sci..

[10] Pfeffer S.R. (2017). Rab GTPases: master regulators that establish the secretory and endocytic pathways. Mol. Biol. Cell.

[11] Homma Y., Hiragi S., Fukuda M. (2021). Rab family of small GTPases: an updated view on their regulation and functions. FEBS J..

[12] Huber L.A., Pimplikar S., Parton R.G., Virta H., Zerial M., Simons K. (1993). Rab8, a small GTPase involved in vesicular traffic between the TGN and the basolateral plasma membrane. J. Cell Biol*.*.

[13] Peränen J., Auvinen P., Virta H., Wepf R., Simons K. (1996). Rab8 promotes polarized membrane transport through reorganization of actin and microtubules in fibroblasts. J. Cell Biol..

[14] Hattula K., Furuhjelm J., Tikkanen J., Tanhuanpää K., Laakkonen P., Peränen J. (2006). Characterization of the Rab8-specific membrane traffic route linked to protrusion formation. J. Cell Sci..

[15] Henry L., Sheff D.R. (2008). Rab8 regulates basolateral secretory, but not recycling, traffic at the recycling endosome. Mol. Biol. Cell.

[16] Sharma M., Giridharan S.S.P., Rahajeng J., Naslavsky N., Caplan S. (2009). MICAL-L1 links EHD1 to tubular recycling endosomes and regulates receptor recycling. Mol. Biol. Cell.

[17] Peränen J. (2011). Rab8 GTPase as a regulator of cell shape. Cytoskeleton.

[18] Merithew E., Hatherly S., Dumas J.J., Lawe D.C., Heller-Harrison R., Lambright D.G. (2001). Structural plasticity of an invariant hydrophobic triad in the switch regions of Rab GTPases is a determinant of effector recognition. J. Biol. Chem..

[19] Eathiraj S., Pan X., Ritacco C., Lambright D.G. (2005). Structural basis of family-wide Rab GTPase recognition by rabenosyn-5. Nature.

[20] Grosshans B.L., Ortiz D., Novick P. (2006). Rabs and their effectors: achieving specificity in membrane traffic. Proc. Natl. Acad. Sci. USA.

[21] Pfeffer S., Aivazian D. (2004). Targeting Rab GTPases to distinct membrane compartments. Nat. Rev. Mol. Cell Biol..

[22] Blümer J., Rey J., Dehmelt L., Mazel T., Wu Y.W., Bastiaens P., Goody R.S., Itzen A. (2013). RabGEFs are a major determinant for specific Rab membrane targeting. J. Cell Biol..

[23] Rybin V., Ullrich O., Rubino M., Alexandrov K., Simon I., Seabra M.C., Goody R., Zerial M. (1996). GTPase activity of Rab5 acts as a timer for endocytic membrane fusion. Nature.

[24] Nuoffer C., Wu S.K., Dascher C., Balch W.E. (1997). Mss4 does not function as an exchange factor for Rab in endoplasmic reticulum to Golgi transport. Mol. Biol. Cell.

[25] Chen W., Feng Y., Chen D., Wandinger-Ness A. (1998). Rab11 is required for trans-golgi network-to-plasma membrane transport and a preferential target for GDP dissociation inhibitor. Mol. Biol. Cell.

[26] Chen X., Edwards J.A.S., Logsdon C.D., Ernst S.A., Williams J.A. (2002). Dominant negative Rab3D inhibits amylase release from mouse pancreatic acini. J. Biol. Chem..

[27] Nokes R.L., Fields I.C., Collins R.N., Fölsch H. (2008). Rab13 regulates membrane trafficking between TGN and recycling endosomes in polarized epithelial cells. J. Cell Biol..

[28] Cardoso C.M.P., Jordao L., Vieira O.V. (2010). Rab10 regulates phagosome maturation and its overexpression rescues Mycobacterium-containing phagosomes maturation. Traffic.

[29] Mizuno-Yamasaki E., Rivera-Molina F., Novick P. (2012). GTPase networks in membrane traffic. Annu. Rev. Biochem..

[30] Li H., Ou L., Fan J., Xiao M., Kuang C., Liu X., Sun Y., Xu Y. (2017). Rab8A regulates insulin-stimulated GLUT4 translocation in C2C12 myoblasts. FEBS Lett..

[31] Nachury M.V., Loktev A.V., Zhang Q., Westlake C.J., Peränen J., Merdes A., Slusarski D.C., Scheller R.H., Bazan J.F., Sheffield V.C., Jackson P.K. (2007). A core complex of BBS proteins cooperates with the GTPase Rab8 to promote ciliary membrane biogenesis. Cell.

[32] Yoshimura S.I., Egerer J., Fuchs E., Haas A.K., Barr F.A. (2007). Functional dissection of Rab GTPases involved in primary cilium formation. J. Cell Biol..

[33] Minami S., Yokota N., Kawahara H. (2020). BAG6 contributes glucose uptake by supporting the cell surface translocation of the glucose transporter GLUT4. Biol. Open.

[34] Hattula K., Furuhjelm J., Arffman A., Peränen J. (2002). A Rab8-specific GDP/GTP exchange factor is involved in actin remodeling and polarized membrane transport. Mol. Biol. Cell.

[35] Takahashi T., Minami S., Tsuchiya Y., Tajima K., Sakai N., Suga K., Hisanaga S.I., Ohbayashi N., Fukuda M., Kawahara H. (2019). Cytoplasmic control of Rab-family small GTPases through BAG6. EMBO Rep..

[36] Kang S.W., Rane N.S., Kim S.J., Garrison J.L., Taunton J., Hegde R.S. (2006). Substrate-specific translocational attenuation during ER stress defines a pre-emptive quality control pathway. Cell.

[37] Minami R., Hayakawa A., Kagawa H., Yanagi Y., Yokosawa H., Kawahara H. (2010). BAG6 is essential for selective elimination of defective proteasomal substrates. J. Cell Biol..

[38] Hessa T., Sharma A., Mariappan M., Eshleman H.D., Gutierrez E., Hegde R.S. (2011). Protein targeting and degradation pathway are coupled for elimination of misfolded proteins. Nature.

[39] Wang Q., Liu Y., Soetandyo N., Baek K., Hegde R., Ye Y. (2011). A ubiquitin ligase-associated chaperone holdase maintains polypeptides in soluble states for proteasome degradation. Mol. Cell.

[40] Kawahara H., Minami R., Yokota N. (2013). BAG6/BAT3; emerging roles in quality control for nascent polypeptides. J. Biochem..

[41] Lee J.G., Ye Y. (2013). Bag6/Bat3/Scythe: a novel chaperone activity with diverse regulatory functions in protein biogenesis and degradation. Bioessays.

[42] Guna A., Hegde R.S. (2018). Transmembrane domain recognition during membrane protein biogenesis and quality control. Curr. Biol..

[43] Kikukawa Y., Minami R., Shimada M., Kobayashi M., Tanaka K., Yokosawa H., Kawahara H. (2005). Unique proteasome subunit Xrpn10c is a specific receptor for the antiapoptotic ubiquitin-like protein Scythe. FEBS J..

[44] Tanaka H., Takahashi T., Xie Y., Minami R., Yanagi Y., Hayashishita M., Suzuki R., Yokota N., Shimada M., Mizushima T. (2016). A conserved island of BAG6/Scythe is related to ubiquitin domains and participates in short hydrophobicity recognition. FEBS J..

[45] Rodrigo-Brenni M.C., Gutierrez E., Hegde R.S. (2014). Cytosolic quality control of mislocalized proteins requires RNF126 recruitment to Bag6. Mol. Cell.

[46] Hu X., Wang L., Wang Y., Ji J., Li J., Wang Z., Li C., Zhang Y., Zhang Z.R. (2020). RNF126-mediated reubiquitination is required for proteasomal degradation of p97-extracted membrane proteins. Mol. Cell.

[47] Suzuki R., Kawahara H. (2016). UBQLN4 recognizes mislocalized transmembrane domain proteins and targets these to proteasomal degradation. EMBO Rep..

[48] Saeki Y. (2017). Ubiquitin recognition by the proteasome. J. Biochem..

[49] Sato T., Iwano T., Kunii M., Matsuda S., Mizuguchi R., Jung Y., Hagiwara H., Yoshihara Y., Yuzaki M., Harada R., Harada A. (2014). Rab8a and Rab8b are essential for several apical transport pathways but insufficient for ciliogenesis. J. Cell Sci..

[50] Zheng T., Yang Y., Castañeda C.A. (2020). Structure, dynamics and functions of UBQLNs: at the crossroads of protein quality control machinery. Biochem. J..

[51] Stroupe C., Brunger A.T. (2000). Crystal structures of a Rab protein in its inactive and active conformations. J. Mol. Biol..

[52] Guo Z., Hou X., Goody R.S., Itzen A. (2013). Intermediates in the guanine nucleotide exchange reaction of Rab8 protein catalyzed by guanine nucleotide exchange factors Rabin8 and GRAB. J. Biol. Chem..

[53] Homma Y., Fukuda M. (2016). Rabin8 regulates neurite outgrowth in both GEF activity-dependent and -independent manners. Mol. Biol. Cell.

[54] Kaiho-Soma A., Akizuki Y., Igarashi K., Endo A., Shoda T., Kawase Y., Demizu Y., Naito M., Saeki Y., Tanaka K., Ohtake F. (2021). TRIP12 promotes small-molecule-induced degradation through K29/K48-branched ubiquitin chains. Mol. Cell.

[55] Lu X., Liu S., Kornberg T.B. (2006). The C-terminal tail of the Hedgehog receptor Patched regulates both localization and turnover. Genes Dev..

[56] Singla V., Reiter J.F. (2006). The primary cilium as the cell’s antenna: signaling at a sensory organelle. Science.

[57] Blacque O.E., Scheidel N., Kuhns S. (2018). Rab GTPases in cilium formation and function. Small GTPases.

[58] Knödler A., Feng S., Zhang J., Zhang X., Das A., Peränen J., Guo W. (2010). Coordination of Rab8 and Rab11 in primary ciliogenesis. Proc. Natl. Acad. Sci. USA.

[59] Feng S., Knödler A., Ren J., Zhang J., Zhang X., Hong Y., Huang S., Peränen J., Guo W. (2012). A Rab8 guanine nucleotide exchange factor-effector interaction network regulates primary ciliogenesis. J. Biol. Chem..

[60] He X., Zhang Y., Yang L., Feng J., Yang S., Li T., Zhong T., Li Q., Xie W., Liu M. (2020). BAG6 is a novel microtubule-binding protein that regulates ciliogenesis by modulating the cell cycle and interacting with γ-tubulin. Exp. Cell Res..

[61] Mariappan M., Li X., Stefanovic S., Sharma A., Mateja A., Keenan R.J., Hegde R.S. (2010). A ribosome-associating factor chaperones tail-anchored membrane proteins. Nature.

[62] Leznicki P., Clancy A., Schwappach B., High S. (2010). Bat3 promotes the membrane integration of tail-anchored proteins. J. Cell Sci..

[63] Krysztofinska E.M., Martínez-Lumbreras S., Thapaliya A., Evans N.J., High S., Isaacson R.L. (2016). Structural and functional insights into the E3 ligase, RNF126. Sci. Rep..

[64] Kamikubo K., Kato H., Kioka H., Yamazaki S., Tsukamoto O., Nishida Y., Asano Y., Imamura H., Kawahara H., Shintani Y., Takashima S. (2019). A molecular triage process mediated by RING finger protein 126 and BCL2-associated athanogene 6 regulates degradation of G _0_/G _1_ switch gene 2. J. Biol. Chem..

[65] Itzen A., Pylypenko O., Goody R.S., Alexandrov K., Rak A. (2006). Nucleotide exchange via local protein unfolding-structure of Rab8 in complex with MSS4. EMBO J..

[66] Smith C.J., Berry D.M., McGlade C.J. (2013). The E3 ubiquitin ligases RNF126 and Rabring7 regulate endosomal sorting of the epidermal growth factor receptor. J. Cell Sci..

[67] Smith C.J., McGlade C.J. (2014). The ubiquitin ligase RNF126 regulates the retrograde sorting of the cation-independent mannose 6-phosphate receptor. Exp. Cell Res..

[68] Zhang Z.D., Li H.X., Gan H., Tang Z., Guo Y.Y., Yao S.Q., Liuyu T., Zhong B., Lin D. (2022). RNF115 inhibits the post-ER trafficking of TLRs and TLRs-mediated immune responses by catalyzing K11-linked ubiquitination of RAB1A and RAB13. Adv. Sci..

[69] Haycraft C.J., Banizs B., Aydin-Son Y., Zhang Q., Michaud E.J., Yoder B.K. (2005). Gli2 and Gli3 localize to cilia and require the intraflagellar transport protein polaris for processing and function. PLoS Genet..

[70] Lobo G.P., Fulmer D., Guo L., Zuo X., Dang Y., Kim S.H., Su Y., George K., Obert E., Fogelgren B. (2017). The exocyst is required for photoreceptor ciliogenesis and retinal development. J. Biol. Chem..

[71] Gerdes J.M., Davis E.E., Katsanis N. (2009). The vertebrate primary cilium in development, homeostasis, and disease. Cell.

[72] Marshall W.F. (2008). The cell biological basis of ciliary disease. J. Cell Biol..

[73] Miyauchi M., Matsumura R., Kawahara H. (2023). BAG6 supports stress fiber formation by preventing the ubiquitin-mediated degradation of RhoA. Mol. Biol. Cell.

[74] Kim J., Lee J.E., Heynen-Genel S., Suyama E., Ono K., Lee K., Ideker T., Aza-Blanc P., Gleeson J.G. (2010). Functional genomic screen for modulators of ciliogenesis and cilium length. Nature.

[75] Stenmark H., Olkkonen V.M. (2001). The Rab GTPase family. Genome Biol..

[76] Noguchi A., Adachi S., Yokota N., Hatta T., Natsume T., Kawahara H. (2018). ZFP36L2 is a cell cycle-regulated CCCH protein necessary for DNA lesion-induced S-phase arrest. Biol. Open.

