# Supplementary material for: Protein quality control machinery supports primary ciliogenesis by eliminating GDP-bound Rab8-family GTPases
Source: iScience. 2023 Apr 10;26(5):106652. doi: 10.1016/j.isci.2023.106652 (PMC10173616; doi:10.1016/j.isci.2023.106652)
Supplement: Document S1. Figures S1–S8 [file mmc1.pdf]

iScience, Volume *26*

Supplemental information

Protein quality control machinery supports primary ciliogenesis by eliminating GDP-bound Rab8-family GTPases

Toshiki Takahashi, Jun Shirai, Miyo Matsuda, Sae Nakanaga, Shin Matsushita, Kei Wakita, Mizuki Hayashishita, Rigel Suzuki, Aya Noguchi, Naoto Yokota, and Hiroyuki Kawahara

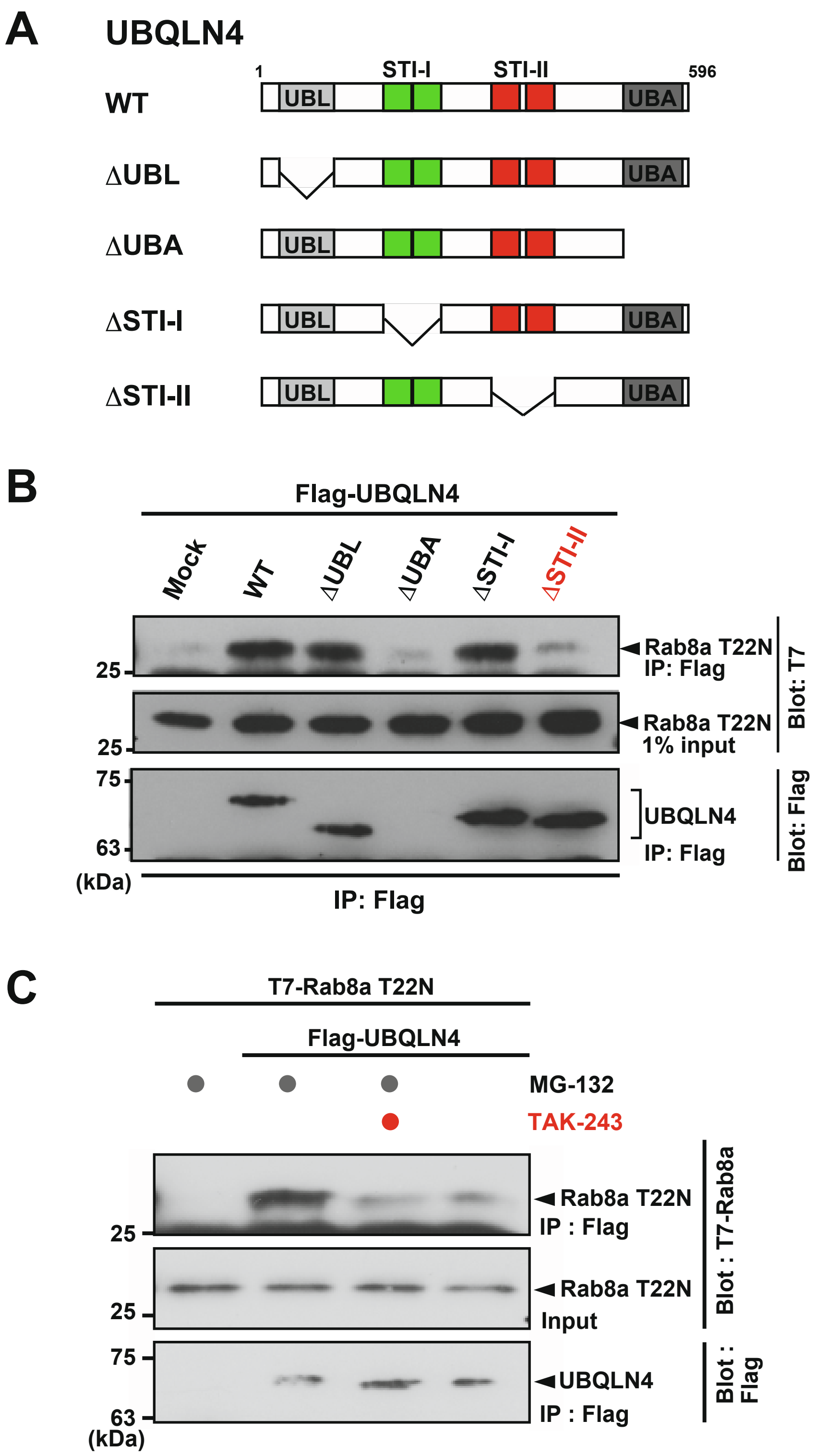

Figure S1.

Related to Figure 2, the STI-II region of UBQLN4 is necessary for binding to GDP-bound Rab8a.

(**A**) Schematic representation of the UBQLN4 deletion mutant proteins used in this study.

(**B**) The wild-type (WT) form of Flag-tagged UBQLN4 and its truncated derivatives were expressed in HeLa cells with T7-tagged Rab8a-T22N. At 4 h after the addition of 10 μM MG-132, UBQLN4 was immunoprecipitated with an anti-Flag antibody and the precipitates were blotted with the antibodies indicated. Note that ΔUBA UBQLN4 protein was difficult to express adequately.

(**C**) Co-precipitation of T7-Rab8a-T22N with Flag-UBQLN4 was weakened by treatment with TAK-243 (MLN7243), an inhibitor for the E1 ubiquitin activating enzyme. TAK-243 was added to the cell cultures at 10 μM for 4 h before cell harvest.

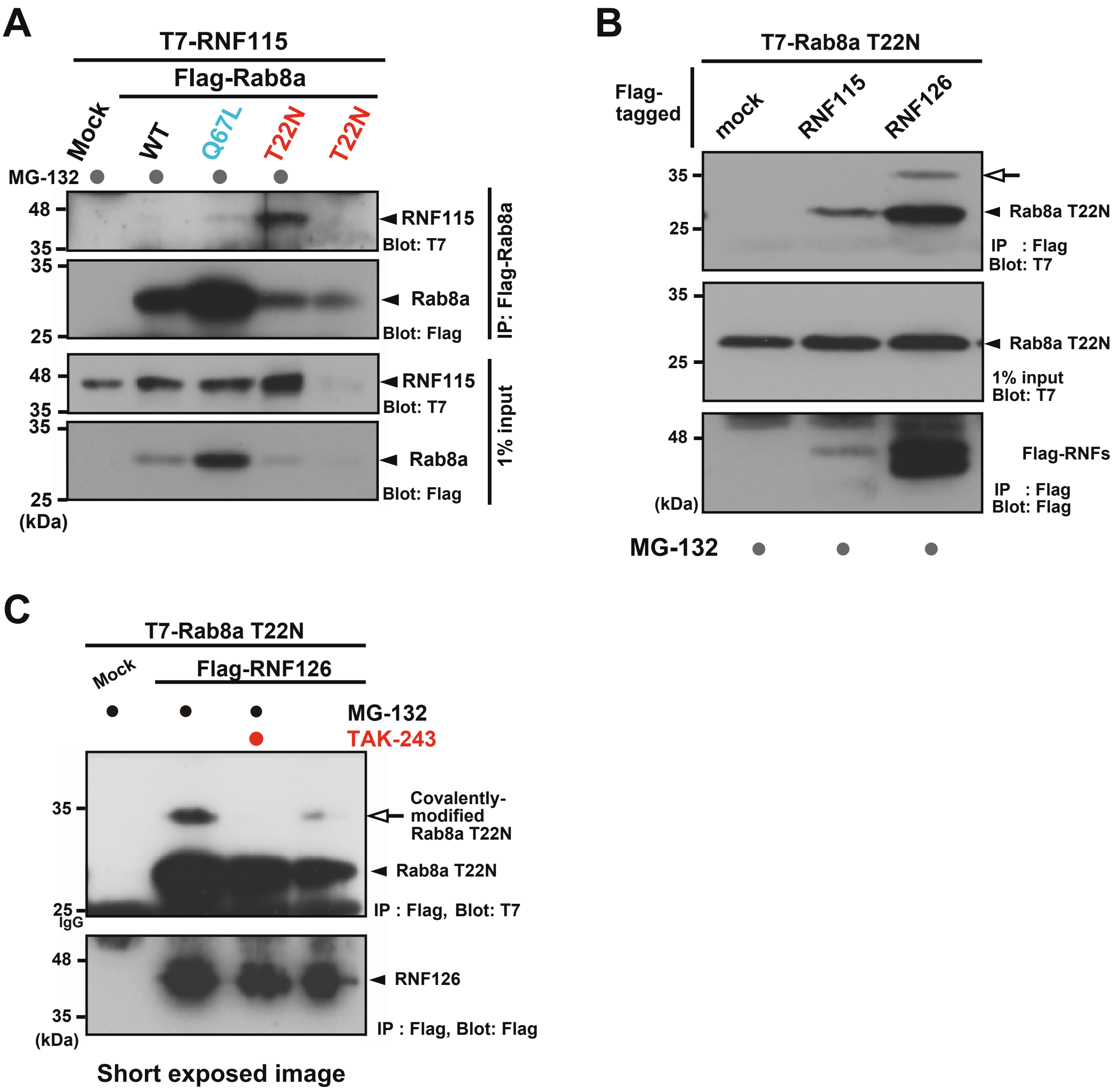

Figure S2.

Related to Figure 4, RNF126 and RNF115 co-precipitated covalently-modified Rab8a.

(**A**) T7-tagged RNF115, a paralog of RNF126, preferentially co-precipitated with Flag-tagged Rab8a-T22N, and less efficiently co-precipitated with Rab8a-Q67L, a constitutively active mutant. MG-132 (10 μM) was added to the cell culture 4 h before harvesting. Note that the Rab8a-T22N was expressed at lower levels than either WT or Q67L due to its instability (see Flag-blot panels).

(**B**) Either Flag-tagged RNF126 or RNF115 was co-expressed with T7-tagged Rab8a-T22N with MG-132 (10 μM) for 4 h before harvesting. These RING-finger E3 ligases associated with Rab8a-T22N. The T7-blot in Flag-RNF126 precipitates showed an additional band of co-precipitated Rab8a-T22N at approximately 35-kDa (indicated as a white arrow). Note that RNF115 is an unstable protein that is difficult to express at a level equivalent to that of RNF126 (see Flag-blot panel).

(**C**) In relation to Figure 4E, a 35-kDa covalent modification of Rab8a-T22N (indicated by a white arrow) co-precipitated with RNF126 was abolished by treatment with 10 μM TAK-243. IgG indicates an immunoglobulin signal.

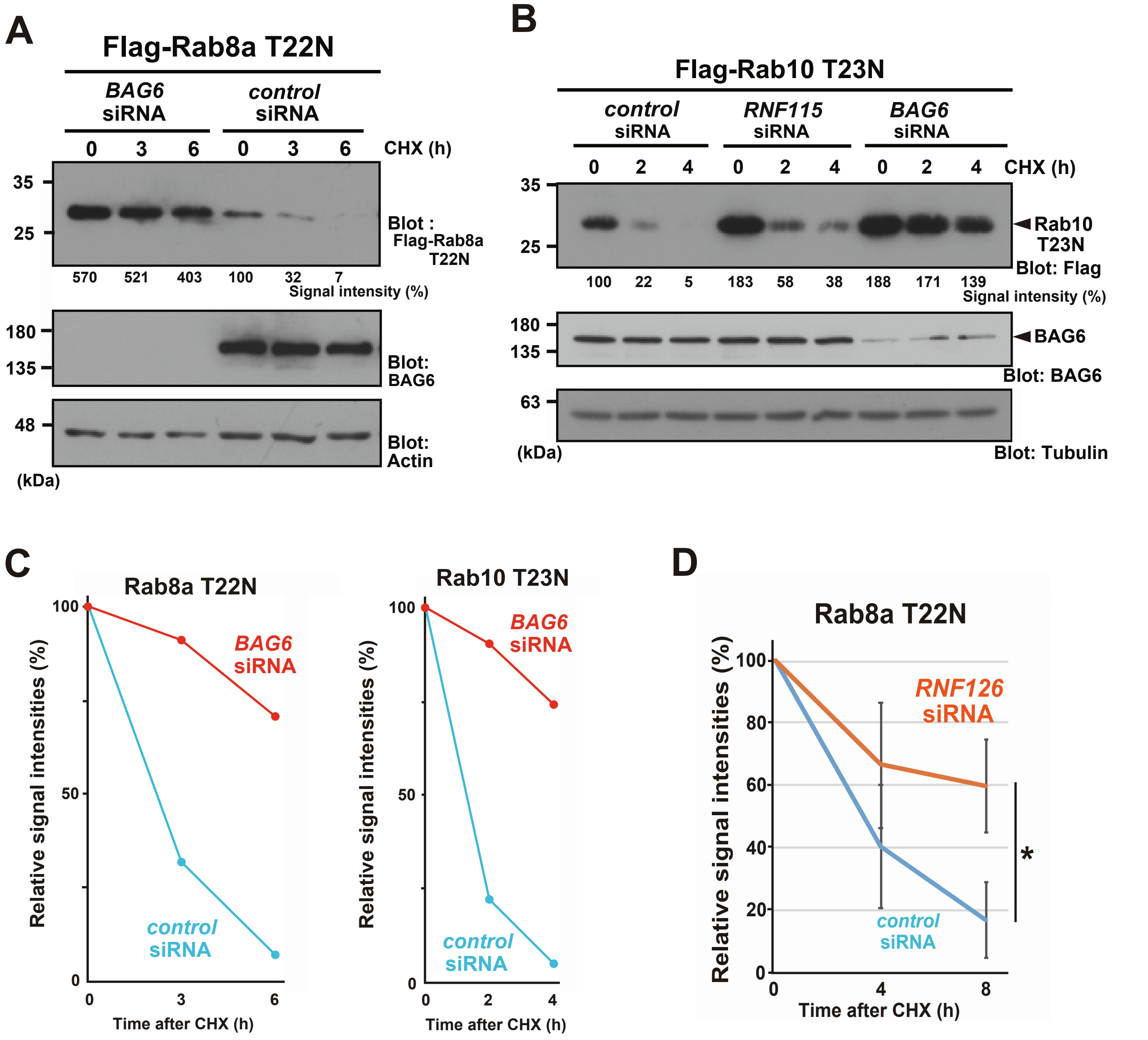

Figure S3.

Related to Figure 5A, endogenous BAG6 is required for the degradation of GDP-bound forms of Rab8a and Rab10 proteins.

(**A, B**) HeLa cells were transfected with 5 nM siRNA duplexes of *BAG6, RNF115*, or *control* siRNA. At 24 h after siRNA transfection, Flag-tagged Rab8a-T22N (A) or Rab10-T23N (B) were expressed in the cells. At 24 h after transfection with Rab-expression vectors, the cells were chased with 20 μg/mL cycloheximide (CHX) and harvested at the indicated time after CHX addition. Endogenous actin or tubulin blots were used as loading controls. Quantified signal intensities of Rab8a (A) and Rab10 (B) protein blots at the indicated time points relative to loading controls were noted at the bottom of the blot panels. The signal at time zero in *control* siRNA-treated cells was defined as 100%.

(**C, D**) Graphs indicate the quantified signal intensities of the Rab8a-T22N (A) and Rab10-T23N (B) protein blots relative to loading controls at the indicated time points after CHX addition (C). RNF126 depletion shown in Figure 5C were performed three independent biological replicates (D). *$p < 0.05$ (Student’ s t-test). N= 3. Results of control-depleted cells are shown as blue lines. Signals at time zero were defined as 100%.

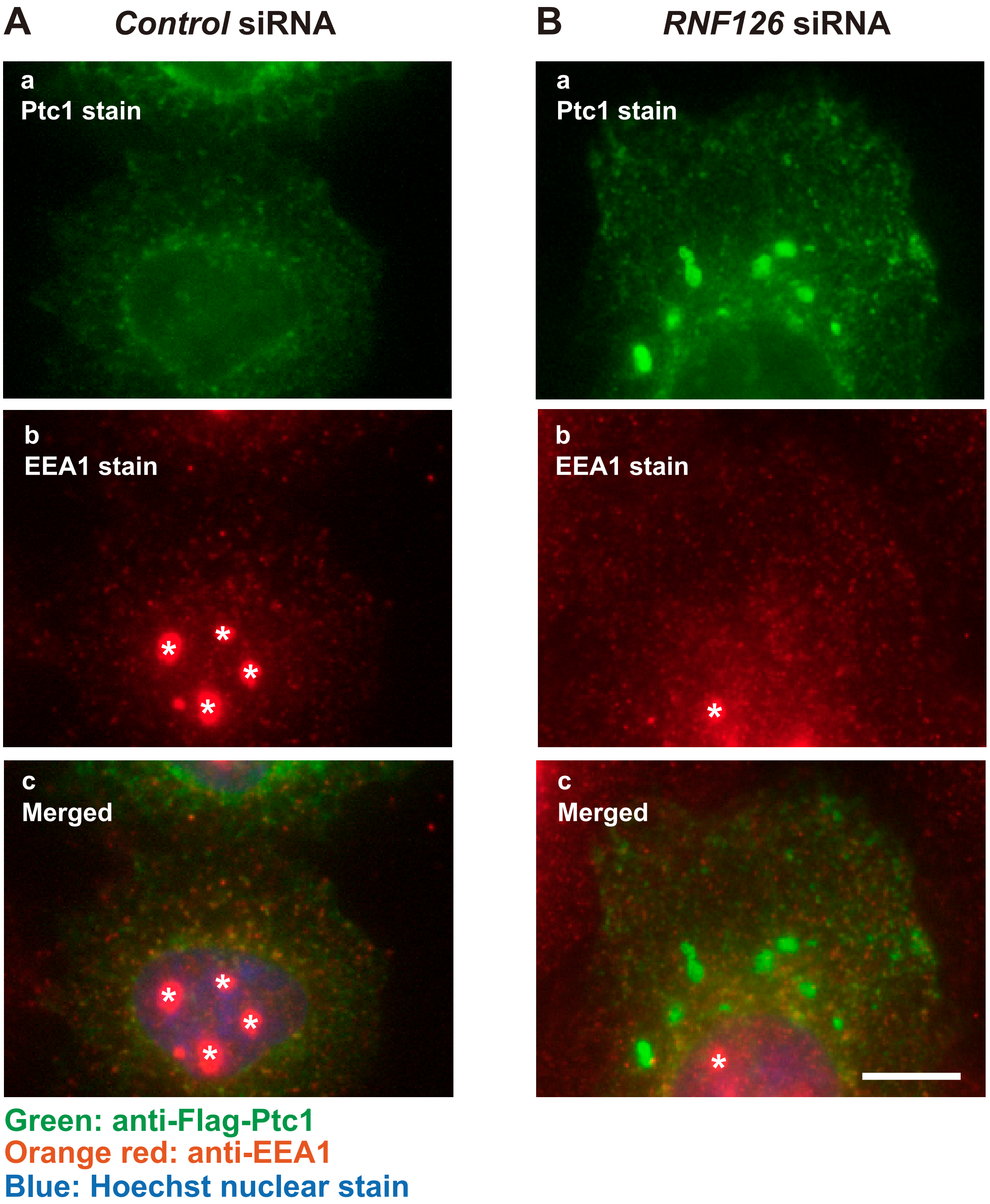

Figure S4.

Related to Figure 6C, co-immunostaining of Ptc1 with endosomal marker protein EEA1.

At 72 h after transfection with siRNA duplexes for *control* and *RNF126* (5 nM each), the intracellular distribution of Patched1 (Ptc1)-Flag was examined (shown in green) with the early endosome marker EEA1 (shown in red). Nuclei were stained with Hoechst 33258 (shown in blue). We observed no overt difference in EEA1 distribution in RNF126-depleted cells compared with the control. Note that we used a pCI-neo-based modified expression plasmid with largely compromised promoter activity to maintain the expression of Flag-tagged Ptc1 protein (Ptc1-Flag) at nearly physiological levels. Asterisks indicate non-specific staining of anti-EEA1 antibody in the nucleus. Scale bar: 10 μm.

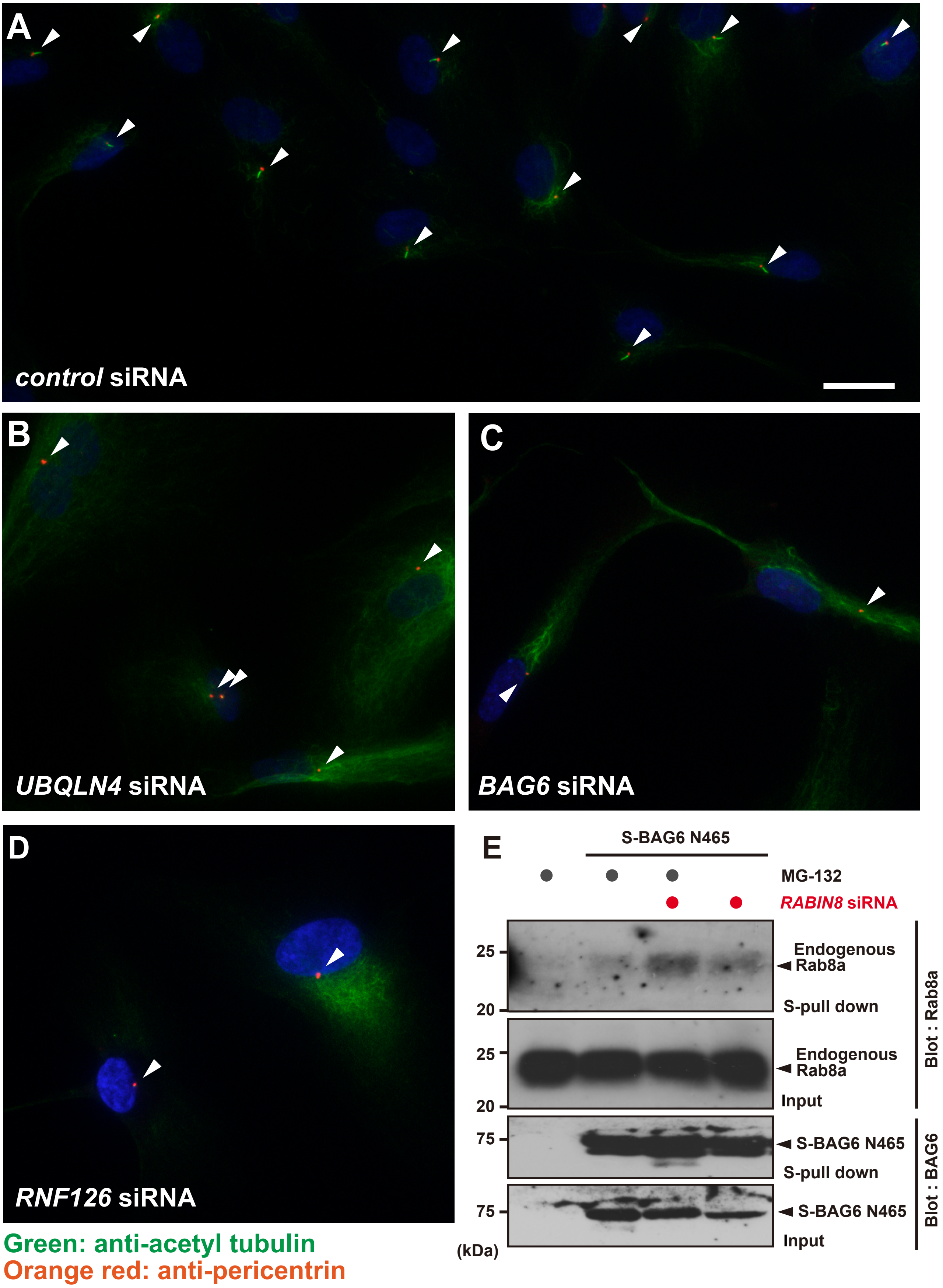
A
control siRNA
B
UBQLN4 siRNA
C
BAG6 siRNA
D
RNF126 siRNA
E
S-BAG6 N465
MG-132
RABIN8 siRNA
25
20
25
20
75
75
(kDa)
Endogenous Rab8a
S-pull down
Endogenous Rab8a
Input
S-BAG6 N465
S-pull down
S-BAG6 N465
Input
Blot : Rab8a
Blot : BAG6
Green: anti-acetyl tubulin
Orange red: anti-pericentrin

Figure S5.

Related to Figure 7B, wide-area view of primary cilia formation in RPE1 cells treated with depletion of pre-emptive quality control machinery components.

(**A-D**) Serum-starved hTERT-RPE1 cells were immunostained with acetylated α-tubulin (a cilial axoneme marker, green) and pericentrin (a basal body marker, red) in siRNA treatments for pre-emptive quality machinery components. The positions of the mother centriole/basal body are indicated by a white arrowhead. Control cells grew primary cilia (rods in the green channel), whereas the cells with BAG6, UBQLN4, and RNF126 depletions failed to grow cilial axonemes on the basal body. Nuclei were stained with Hoechst 33258 (shown in blue). Note that all images presented in this figure were acquired in an identical set of experiments, and the exposure times of all photographs in this figure were the same. Scale bar: 10 μm.

(**E**) Endogenous Rab8a protein was co-precipitated with an S-tagged BAG6 N465 fragment (a probe for GDP-bound Rab8a) from RPE1 cell lysates. Note that both MG-132 treatment and RABIN8 depletion resulted in augmentation of BAG6 co-precipitation with endogenous Rab8a, suggesting that the GDP-bound form of endogenous Rab8a is a target for proteasomal degradation.

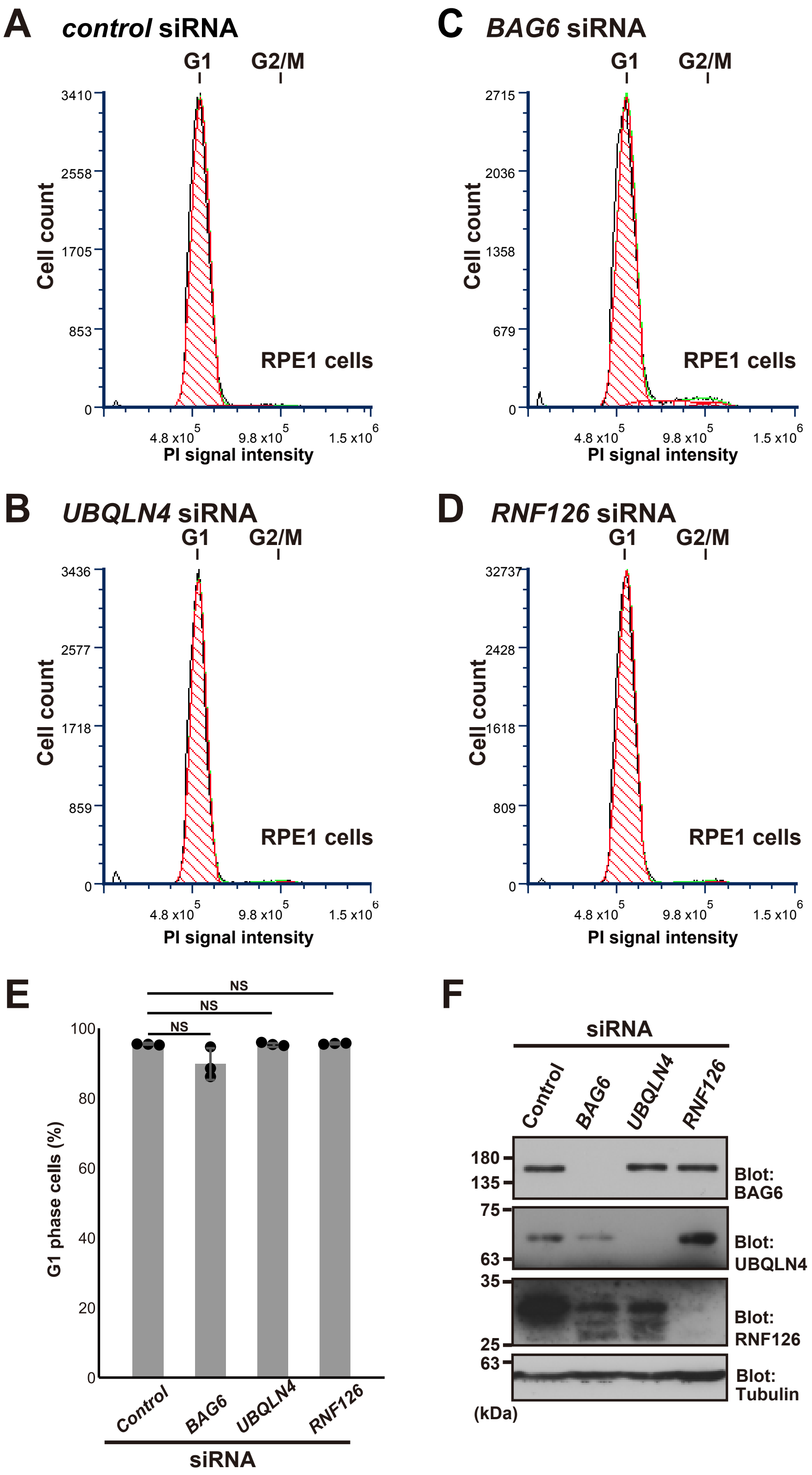

Figure S6.

Related to Figure 7, depletion of RNF126 and UBQLN4 did not alter the G1 population of serum-starved RPE1 cells.

(**A-D**) Flow cytometric analyses of cell cycle distribution in serum-starved hTERT-RPE1 cells. After 24 h of transfection with *control* siRNA (A), *UBQLN4* siRNA (B), *BAG6* siRNA (C), and *RNF126* siRNA (D), the cells were treated with serum starvation for 24 h. The cells were harvested and their cell cycle profiles were analyzed using a flow cytometer with propidium iodide (PI) staining. (**E**) Cell cycle distribution data are quantified and shown as means ± S.D. calculated from three independent biological replicates. NS; not significant. (**F**) Efficacies of knockdown were verified by immunoblot analysis. Total cell lysates from hTERT-RPE1 cells treated with siRNAs were immunoblotted with the respective antibodies for BAG6, UBQLN4, RNF126, and tubulin (loading control).

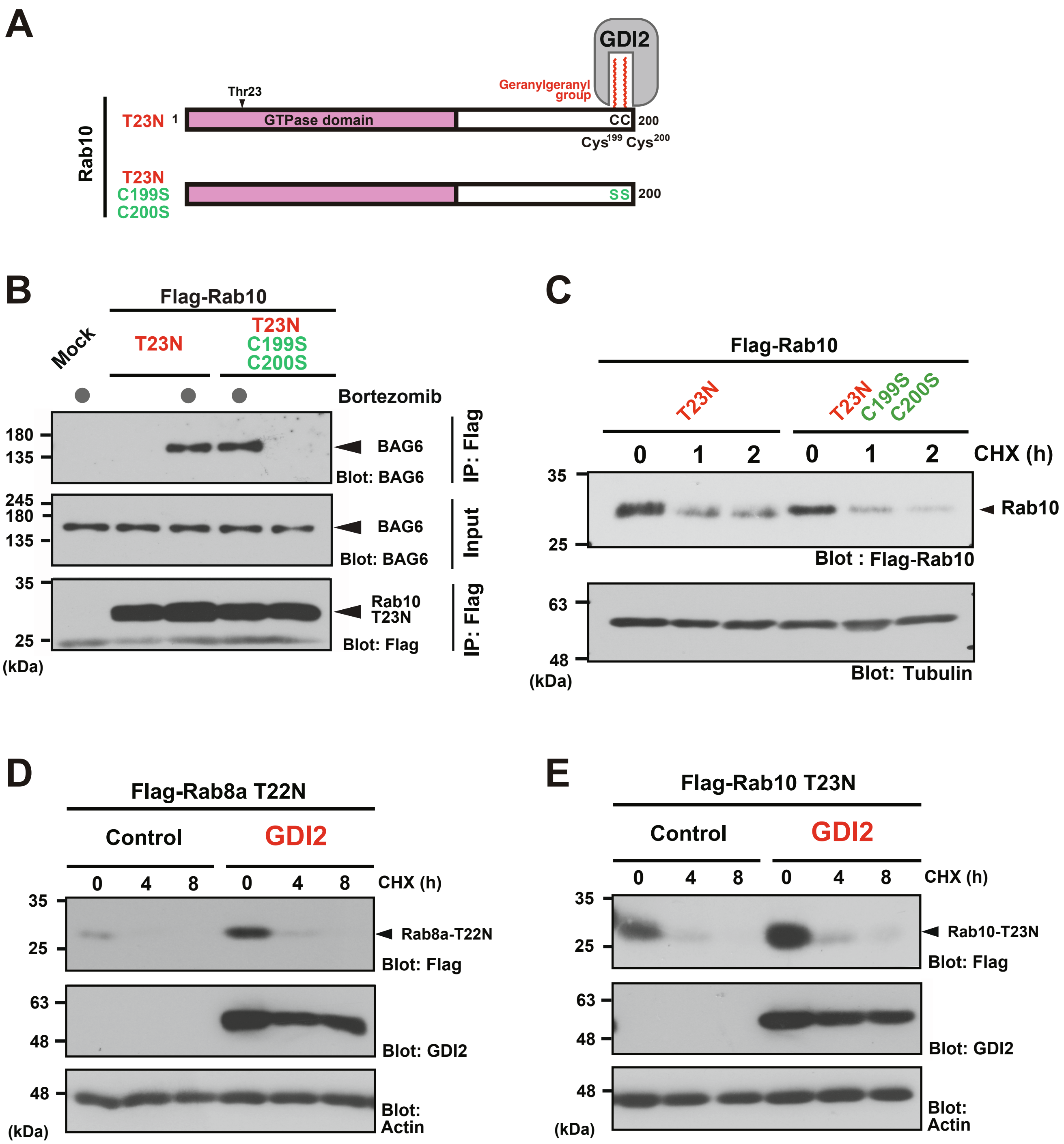

Figure S7.

Related to Figure 8 and Discussion, C-terminal geranylgeranylations of Rab8a and Rab10 are not important for the instability of their GDP-bound forms.

(**A**) Schematic representation of the GDP-bound form of Rab10 protein (T23N). To prevent C-terminal geranylgeranylation, the Cys199 and Cys200 residues were substituted with serine and designated as C199S/C200S.

(**B, C**) C-terminal geranylgeranylations of Rab10-T23N were not required for either BAG6 interaction (B) or instability in the cytoplasm (C).

(**D, E**) Shortage of GDI is not the cause of instabilities in GDP-bound Rab8a and Rab10. Forced expression of GDI2, a cytoplasmic chaperone of isoprenylated Rab proteins, did not stabilize Rab8a-T22N (D) and Rab10-T23N (E) in HeLa cells.

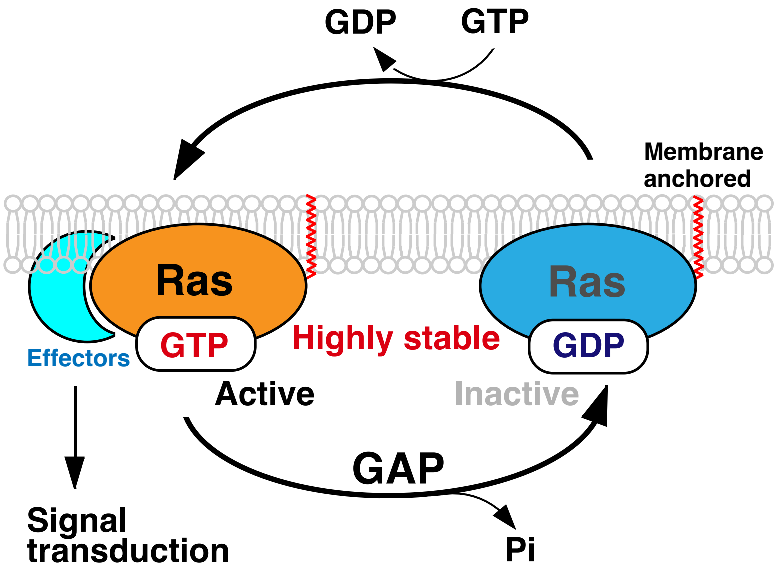

Figure S8.

Related to Figure 8, general principle of Ras activation on the cell membrane.

Ras is a cell-membrane-anchored molecular switch cycling between active GTP-bound and inactive GDP-bound states. GDP/GTP exchange is stimulated by guanine nucleotide exchange factor (GEF) to transduce proliferative signals in response to extracellular growth stimuli. In the absence of growth factors, Ras-bounded GTP is hydrolyzed by intrinsic GTPase activity of Ras, resulting in its rapid inactivation.
